# Potential of siRNA in COVID-19 therapy: Emphasis on *in silico* design and nanoparticles based delivery

**DOI:** 10.3389/fbioe.2023.1112755

**Published:** 2023-02-06

**Authors:** Rushikesh Fopase, Chinmaya Panda, Amarnath P. Rajendran, Hasan Uludag, Lalit M. Pandey

**Affiliations:** ^1^ Bio-Interface & Environmental Engineering Lab, Department of Biosciences and Bioengineering, Indian Institute of Technology Guwahati, Assam, India; ^2^ Department of Chemical & Materials Engineering, Faculty of Engineering, University of Alberta, Edmonton, AB, Canada; ^3^ Faculty of Pharmacy and Pharmaceutical Sciences, University of Alberta, Edmonton, AB, Canada; ^4^ Department of Biomedical Engineering, Faculty of Medicine and Dentistry, University of Alberta, Edmonton, AB, Canada

**Keywords:** siRNA, lipid nanoparticles, surface modification, ligands, COVID-19

## Abstract

Small interfering RNA (siRNA)-mediated mRNA degradation approach have imparted its eminence against several difficult-to-treat genetic disorders and other allied diseases. Viral outbreaks and resulting pandemics have repeatedly threatened public health and questioned human preparedness at the forefront of drug design and biomedical readiness. During the recent pandemic caused by the SARS-CoV-2, mRNA-based vaccination strategies have paved the way for a new era of RNA therapeutics. RNA Interference (RNAi) based approach using small interfering RNA may complement clinical management of the COVID-19. RNA Interference approach will primarily work by restricting the synthesis of the proteins required for viral replication, thereby hampering viral cellular entry and trafficking by targeting host as well as protein factors. Despite promising benefits, the stability of small interfering RNA in the physiological environment is of grave concern as well as site-directed targeted delivery and evasion of the immune system require immediate attention. In this regard, nanotechnology offers viable solutions for these challenges. The review highlights the potential of small interfering RNAs targeted toward specific regions of the viral genome and the features of nanoformulations necessary for the entrapment and delivery of small interfering RNAs. *In silico* design of small interfering RNA for different variants of SARS-CoV-2 has been discussed. Various nanoparticles as promising carriers of small interfering RNAs along with their salient properties, including surface functionalization, are summarized. This review will help tackle the real-world challenges encountered by the *in vivo* delivery of small interfering RNAs, ensuring a safe, stable, and readily available drug candidate for efficient management of SARS-CoV-2 in the future.

## Introduction

RNAi therapeutics have had a promising impact in reducing the expression of disease-associated genes ever since their discovery in 1990s (Kalita et al., 2022). The field received its major breakthrough in 2018 with the approval of the first siRNA-based drug ‘Patisiran’ (Onpattro^®^) for the treatment of transthyretin-mediated amyloidosis (Zhang et al., 2022a). Since then, over 30 drug candidates have been in the clinical trial pipeline as next-generation medicines to develop medications for difficult-to-treat (‘undruggable’) genetic disorders and ever-evolving SARS-CoV-2-like viral pandemics (Bunea et al., 2020). In this treatment approach, the double-stranded RNAs (dsRNA) designed explicitly against specific disease-causing mRNA sequences are loaded onto a gene regulatory complex, i.e., RNA-induced silencing complex (RISC), consisting of DICER, Argonaute-2 (Ago2), and transactivation response RNA-binding protein (TRBP) proteins (Jayaraman et al., 2012). The dsRNA is cleaved by the RISC complex producing two different strands, out of which the passenger strand is lost, with the guide strand getting paired with the target mRNA meant to be cleaved (Dobrowolski et al., 2021; Ly et al., 2022). Finally, Ago2, the catalytic precursor of the process, cleaves the bound mRNA (Han et al., 2020).

The promising therapeutic benefits of siRNAs are overshadowed by the difficulty in attaining optimal biodistribution and pharmacokinetics of the RNAi therapeutic agents. Various intracellular hurdles, such as non-targeted accumulation in the liver/spleen, impaired long-term protein expression, immunological response, endosomal escape, and post-administration reactions, pose a significant challenge to siRNA therapy (Kalita et al., 2022). To effectively counter such challenges, proper computational approach-based siRNA designing is deemed critical (Idris et al., 2021). Further, the stability and early *in vivo* elimination issues are resolved by loading siRNA molecules onto a suitable nanocarrier targeted against specific tissues or cells (Evers et al., 2022). Nanocarriers tend to improve the overall potency of naked siRNA molecules, reducing the nuclease digestion, off-target binding and unwarranted immune reactions (Gupta et al., 2019). In addition, the surface functionalization of nanoparticles using suitable ligands ensures a site-specific delivery (Khanali et al., 2021).

Severe acute respiratory syndrome coronavirus 2 (SARS-CoV-2) is a pathogenic and transmissible form of coronavirus which emerged in late 2019, creating havoc in the World and posing a major public threat to health and safety (Hu et al., 2021). Novel mRNA-based vaccines such as that from Pfizer-BioNTech (BNT162b2) and Moderna (mRNA-1273), have been quickly developed to restrict the growth of the virus (Corbett et al., 2020; Walsh et al., 2020; Baden et al., 2021). Likewise, to aid the scientific community in developing safe treatment strategies against the virus, drug candidates can also be developed using the concept of siRNA-mediated therapeutics that will utilize the endogenous RNAi pathway. The present review, on one hand, discusses *in silico* strategies for siRNA design against various functional genomic regions of the SARS-CoV-2. On the other hand, select carrier molecules, such as lipid-based and polymeric nanoparticles for siRNA entrapment and delivery are highlighted with their preparation methods since, without an appropriate carrier, RNAi therapeutics display sub-optimal pharmacological activities. The review also briefly explains the targeting of siRNA-loaded lipid nanoparticles (LNPs) and the release of siRNA *in vivo* for desired inhibition. Two comprehensive tables are penned down detailing the siRNA sequences specifically evaluated against SARS-CoV-2 viral genome segments, along with approved siRNA drugs, and ongoing clinical trials. The siRNA completely recognizes and base pairs with the mRNA of interest, followed by its degradation. In contrast, small molecule and antibody-based drugs recognize only a specific protein conformation, making the siRNA robust to address any disease-associated genes. Moreover, siRNA needs to be administered less frequently, in contrast to antibody-based drugs requiring frequent administration. With such advantages come quicker research and developmental span, together with a broader economic and therapeutic perspective (Hu et al., 2020). The review equivocally concludes the significance of siRNA-based nanoparticle formulation as a better alternative against SARS-CoV-2.

## Design of small interfering RNA for different variants of SARS-CoV-2

The single-stranded positive RNA (+ssRNA) genome of the COVID-19 virus, a member of the β-coronavirus family, encodes about 29 proteins out of which four are structural proteins, the spike (S), membrane (M), envelope (E), and nucleocapsid (N) proteins (Muhseen et al., 2020; Thi Nhu Thao et al., 2020; V’kovski et al., 2021). Additionally, the genome has at least five open reading frames (ORFs) with the first ORF (ORF1a/b) occupying 70% of the entire genome. Considering the overall structure of the viral genome to be 5′-UTR-ORF1ab-S-E-M-N-3′-UTR-poly adenine-tail, ORFs constitute nearly two-thirds and are necessary during viral replication. There could broadly be two categories of RNAi targets for coronavirus. The first one involves viral proteins required for growth and replication, whereas the other is related to viral cellular entry and trafficking (Uludağ et al., 2020).

Since ORFs modulate the infectivity of the virus, RNAi-based strategies have been employed against the ORFs. An *in vitro* study designed siRNAs targeted against the ORF1a/b region of the viral genome (coding for a non-structural protein) which reduced the viral burden by 99% and 97% in Vero E6 and Huh-7 hepatoma cells, respectively (Friedrich et al., 2022). A bioinformatics screening study of siRNA libraries on the basis of melting temperature (T_m_), GC content, heat capacity (C_p_), and free energy of hybridization has identified potential therapeutic agents against SARS-CoV-2 including pre-miRNA hairpins and siRNAs (Hasan et al., 2021). The siRNAs were found to target multiple SARS-CoV-2 variants, *viz.* Wuhan-Hu-1 (MN908947.3), alpha (MW686007.1), beta (MW880890), gamma (LR963075.1), delta (MW994451), and omicron (OV112121) (Friedrich et al., 2022). However, this study did not consider any *in vivo* delivery approach. Another *in vitro* study using HEK-293 and Vero E6 cells reported the design of siRNA sequences to target the ORF1 considering the alpha (B.1.1.7 and Q. x), beta (B.1.351, B.1.351.2 and B.1.351.3), gamma (P.1 and P.1. x), delta (B.1.617.2 and AY. x), lambda (C.37 and C.37.1), and mu (B.1.621 and B.1.621.1) variants of the virus. At a concentration of 50 nM, one designed sequence “5′-GGU​ACU​UGG​UAG​UUU​AGC​UTT-3'” inhibited the viral replication by 92.8% (Ambike et al., 2022). However, the study did not consider any *in vivo* delivery approach.

Apart from ORFs, the four structural proteins (S, E, M, and N) have also been identified as RNAi targets (Sajid et al., 2021). Focusing on the spike (S) protein, the receptor-binding domain in the S1 segment binds to the ACE2 receptor of the plasma membrane to enter the host cells and illicit the host immune cell response. Recent siRNA studies on HEK-293 cells and the human primary airway-tracheal cells (hpTCs) have been depicted to reduce the protein expression of spike protein in a dose-dependent manner (Gallicano et al., 2022). The authors imply the use of cholesterol moiety to modify the siRNA to reduce the use of toxic siRNA transfection agents. Lipid-modified siRNAs can also exert equally robust inhibition (Gallicano et al., 2022). Several bioinformatics-based studies have also been reported in regard to the spike protein (Chen et al., 2020; Niktab et al., 2021; Panda et al., 2021; Ayyagari, 2022). However, the spike protein is prone to mutations, as observed in SARS-CoV-2 variants (i.e., alpha (B.1.1.7), beta (B.1.351), gamma (P.1), and delta (B.1.617.2)). Similarly, ACE-2, the receptor for the S-protein also acts as an important siRNA target (Xiao et al., 2020). An *in vitro* approach designed effective siRNAs targeted against the ACE-2 mRNA, reduced the mRNA expression by 90% in 6 days, with 92% viral burden reduction in Vero E6 and Huh-7 hepatoma cells (Friedrich et al., 2022). Whether targeting viral or host targets are more effective (preferable) is an open question.

The viral M protein is responsible for maintaining the structural integrity of the viral membrane and helps bind to nucleocapsids. With the N-terminal ectodomain and C-terminal endodomain, the viral M-protein may act as a suitable siRNA target (Ullah et al., 2020). Likewise, the E protein helps in viral assembly and release, and may also act as siRNA target. As mutations can alter the siRNA sequence thereby reduce its efficiency, hence targeting a strongly conserved region such as the 5′-UTR (untranslated region) is always preferred (Zhang et al., 2022a).

Several *in silico* studies have pioneered the field of siRNA prediction, synthesis and design for SARS-CoV-2. An *in silico* analysis study against the leader sequence of the virus depicted the highest binding score as indicated by the HNADOCK server (Pandey and Verma, 2021). Another *in silico* study designed siRNAs against the viral S-protein, ORF1ab, ORF3a, E-protein, and M-protein (Niktab et al., 2021). Moreover, another group designed siRNA sequences against the RNA-dependent RNA polymerase (RdRp) gene of SARS-CoV-2, and checked their binding scores with the RdRp gene segment by docking and molecular dynamics simulation (Shawan et al., 2021). One of the preliminary *in silico* approaches by Chen et al. predicted 9 siRNA sequences directed against the ORF1a/b, S, ORF3a, M, and the N-protein regions of the viral genome (Chen et al., 2020). The authors also incorporated single point mutations across different variant strains of the virus. In a separate *in silico* study, 139 SARS-CoV-2 sub-strains were considered and found a total of 34 conserved regions (15 in nucleocapsid and 19 in surface glycoprotein) (Chowdhury et al., 2021). The authors have then developed 78 siRNA sequences targeting the surface glycoprotein and nucleocapsid (N) phosphoprotein based on the U, A, and R rules. The authors also modelled the Ago2 and performed molecular docking of siRNAs with Ago2 to find out the best siRNA sequences (Chowdhury et al., 2021).

**FIGURE 1:**
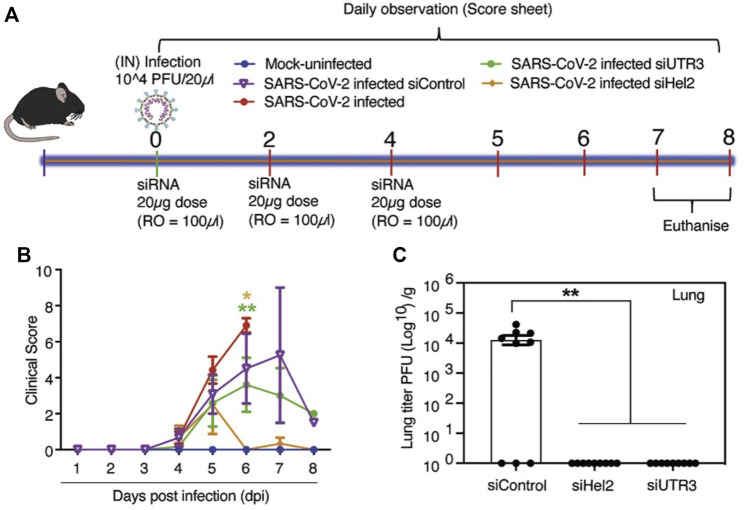
**(A)** Experimental timeline of *in vivo* study in K18-hACE2 mice employing LNP to deliver siRNA. **(B)** Intravenous administered LNP-siRNA formulation improved the clinical score at 6 days post-infection. **(C)** The amount of infectious virus particles in lung tissues at 6 days post-infection as titrated by immunoplaque assays on Vero E6 cells. siRNAs targeted to helicase-2 and UTR3 exhibited decreased or no residual viral particles. Adapted with permissions from Ref. (Idris et al., 2021).

Several *in vitro* and *in vivo* studies have also focused on the screening, modification and delivery of targeted siRNAs for obstructing viral growth. A study combining the computational screening with *in vivo* targeting approach found 13 suitable siRNAs against the viral RdRp and the N protein (Khaitov et al., 2021). The siRNAs, however, were modified with locked nucleic acids (LNAs) for imparting stability and were delivered through a non-toxic peptide dendrimer KK-46 carrier into the destined cells in culture or in Syrian hamsters by inhalation (Khaitov et al., 2021). A daily concentration of ∼3.5 mg/kg of siR-KK-46 reduced lung inflammation as indicated by histopathological microscopic observation on day 6 as compared to the non-treated animals (Khaitov et al., 2021). However, the study did not verify the siRNAs against other prevalent SARS-CoV-2 strains. Another study designed 8 siRNAs targeting the 5′-UTR region of the virus with one molecule inhibiting the replication of SARS-CoV-1 and alpha variant of SARS-CoV-2 at 10 nM of concentration as indicated by *in vitro* studies in Vero E6 cells (Tolksdorf et al., 2021). However, *in vivo* delivery studies were not performed. In one major study (Idris et al., 2021), siRNAs were designed targeting the conserved regions of the virus, i.e., RdRp, helicase, and the 5-UTR. Three sequences were found to reduce viral growth by 90% in Vero E6 cells. The siRNAs were chemically modified with 2′-O-methyl and phosphorothioate to impart stability against the nucleases (Idris et al., 2021). Furthermore, the modified siRNAs were formulated with a delivery vehicle based on LNPs for *in vivo* studies. Intravenous retro-orbital administration of 100 µL of the siRNA-LNP formulation at a concentration of 1 mg/kg in K18-hACE2 mice, restored mice weight and modulated the immune gene expression (Figure 1) (Idris et al., 2021). The formulation targeted against the helicase and UTR3 also improved clinical score at 6 days post-infection, with a reduction in the amount of infectious virus particles as titrated by immunoplaque assay (Idris et al., 2021). An inhalable formulation of siRNA at a concentration of ≤30 mg/mL decreased the viral burden by 96.2% in K18-hACE2-transgenic mice, along with a reduction in associated damage (Chang et al., 2022). The siRNA sequences were designed to target specific regions the leader sequence, RdRp, helicase, S, E, N regions, papain-like protease (PLP), and 3C-like protease of SARS-CoV-2 strains, including Alpha, Delta, Gamma, and Epsilon strains (Chang et al., 2022). After the removal of off-target sequences, a total of 11 sequences was able to reduce the viral load by 99% in Vero E6 cells, even at 10 nM of concentration. The modified siRNA was well tolerated and was not found to induce immune stimulation across the range of 20–75 mg/kg, as verified by mRNA expression of pro-inflammatory cytokines tumor necrosis factor-α (TNF-α), interleukin-6 (IL-6) and interferon- γ (IFN-γ) (Chang et al., 2022).

To cater the needs of the growing bioinformatics research, several databases encompassing the list of sequences, thermodynamic features, GC percentage, target genomic data, possible off-target effects, and applicability against multiple strains have emerged (Dar et al., 2016a; Medeiros et al., 2021). These databases also catalog the toxicity assessment information by checking the *in silico* off-target binding against the human genome, stability, probable structure, chemical modification, and experimental verification information (Dar et al., 2016a; Medeiros et al., 2021). Table 1 summarizes the aforementioned siRNA guide strand sequences along with their target sites, key results and drawbacks of the studies.

**TABLE 1:** siRNA guide strand sequences reported in literature along with their target site, and drawback of the study.

siRNA guide strand sequence	Target site of the virus	Key results	Drawbacks	Ref
5′-GCG​AAA​UAC​CAG​UGG​CUU​A-3′	ORF1	Reduction of the viral burden by 99% and 97% in Vero E6 and Huh-7 hepatoma cells	Lack of *in vivo* approach	Friedrich et al. (2022)
5′-UCAAUAGUCUGAACAACUGGU-3′ 5′-UACCUUUUUAGCUUCUUCCAC-3′ 5′-UGUUUAGCAAGAUUGUGUCCG-3′	ORF1a/b	siRNA selected based on their efficient binding score with target sequence	Only *in silico* prediction-based study	Hasan et al. (2021)
5′-GGU​ACU​UGG​UAG​UUU​AGC​UTT-3′	ORF1	92.8% SARS-CoV-2 viral replication inhibition at 50 nM	Lack of *in vivo* approach	Ambike et al. (2022)
5′-UUC​GUU​UAG​AGA​ACA​GAU​CTT-3′	5′UTR	Viral replication inhibition at 10 nM concentration of siRNA in studies in Vero E6 cells	Lack of *in vivo* delivery approach	Tolksdorf et al. (2021)
5′-GGA​AGG​AAG​UUC​UGU​UGA​ATT-3′	RdRp, and N-protein	siRNA modified with locked nucleic acid and KK-46 dendrimer (3.453 mg/kg concentration) to enhance uptake by inhalation in Syrian hamsters	Effectivity was not checked for other variants	Khaitov et al. (2021)
5′-GUU​UAG​AGA​ACA​GAU​CUA​CAA-3′	Leader sequence	siRNA sequence has the highest docking score with the leader sequence and no off-target binding	No *in vitro* or *in vivo* studies performed and not checked against variants	Pandey and Verma, (2021)
5′-GGA​CAA​GUU​UAA​CCA​CGA​A-3′	ACE2	Reduced mRNA expression by 90% in 6 days, with 92% viral burden reduction in Vero E6 and Huh-7 hepatoma cells	Lack of *in vivo* approach	Friedrich et al. (2022)
5′-GTA​CTT​TCT​TTT​GAA​CTT​CTA​CA-3′	S-protein	siRNA sequences selected based on their efficient binding score with target sequences	No *in vivo* or *in vitro* analysis performed or variants considered	Niktab et al. (2021)
5′-CAA​CAA​AGA​TAG​CAC​TTA​A-3′	ORF1ab			
5′-TCA​TAC​CAC​TTA​TGT​ACA​A-3′	ORF1ab			
5′-CCA​AAA​TCA​TAA​CCC​TCA​AA-3′	ORF3a			
5′-AAA​CCT​TCT​TTT​TAC​GTT​TA-3′	E-protein			
5′-CGA​ACG​CTT​TCT​TAT​TAC​AA-3′	M-protein			
5′-UAG​UAC​UAC​AGA​UAG​AGA​CAC-3′	RdRp	Key siRNA was selected based on the efficient binding score with Ago2 by molecular dynamics simulation	No *in vivo* or *in vitro* experiments performed	Shawan et al. (2021)
5′-UCC​UUC​UUU​AGA​AAC​UAU​ACA-3′	ORF1a/b	siRNA selected based on the binding score	Theoretical *in silico* work	Chen et al. (2020)
5′-UGG​UUU​CAC​UAC​UUU​CUG​UUU-3′	ORF1a/b			
5′-CUU​GAA​GCC​CCU​UUU​CUC​UAU​CUU​U-3′	ORF3a			
5′-UUA​AAA​UAU​AAU​GAA​AAU​GGA-3′	S-protein			
5′-CAA​CUA​UAA​AUU​AAA​CAC​AGA-3′	M-protein			
5′-UUG​AAU​ACA​CCA​AAA​GAU​CAC​AUU-3′	N-protein			
5′-UUU​GUA​UGC​GUC​AAU​AUG​CUU-3′	N-protein Surface glycoprotein	siRNA selected based on the molecular docking score between surface proteins and siRNA sequence	*In silico* work without consideration of *in vitro* or *in vivo* work	Chowdhury et al. (2021)
5′-UAA​UUU​GAC​UCC​UUU​GAG​CAC-3′				
5′-GGU​GGU​GUC​AGU​GUU​AUA​A-3′	S-gene	Four sRNA sequences predicted for the spike protein of the virus	*In silico* prediction, and not much detailed about different strains	Panda et al. (2021)
5′-GCA​AAU​GGC​UUA​UAG​GUU​UAA-3′				
5′-GAG​UUA​CAC​AGA​AUG​UUC​UCU-3′				
5′-CAC​AGA​AUG​UUC​UCU​AUG​AGA-3′				
5′-UGU​AAU​AAG​AAA​GCG​UUC​GUG-3′	M-gene	Six siRNAs found for all three S, M, and N-gene of the virus on basis of *in silico* prediction score	Neither *in vitro* or *in vivo* approach, nor any other variants data mentioned	Ayyagari, (2022)
5′-UAA​UAA​GAA​AGC​GUU​CGU​GAU-3′	M-gene			
5′-UGA​AAU​UUG​GAU​CUU​UGU​CAU-3′	N-gene			
5′-UUU​CUU​AGU​GAC​AGU​UUG​GCC-3′	N-gene			
5′-UAG​AAG​UUU​GAU​AGA​UUC​CUU-3′	S-gene			
5′-UUU​UUG​UCU​UGU​UCA​ACA​GCU-3′	S-gene			
5′-UAU​GGG​UUG​GGA​UUA​UCC​UAA​AUG​T-3′	RdRp	siRNA-HFDM LNP formulation at a concentration of 1 mg/kg in 100 µL administered retro-orbitally in mice reduced viral burden	Consideration of different variant data is absent	Idris et al. (2021)
5′-UGU​UGA​UUC​AUC​ACA​GGG​CUC​AGA​A-3′	Helicase			
5′-GUC​CCU​GGU​UUC​AAC​GAG​AAA​ACA​C-3′	UTR1			
5′-AUA​CCU​UCC​CAG​GUA​ACA​AAC​CAA​C-3′	UTR3			
5′-CUG​UCA​AAC​CCG​GUA​AUU​U-3′	RdRp	Inhalable formulation of siRNA reduced the viral load and damage by 96.2% in mice	Inhalation route was considered, use of a targeted delivery system is not mentioned	Chang et al. (2022)
5′-CAG​CAU​UAA​AUC​ACA​CUA​A-3′	PLP			
5′-GCC​ACU​AGU​CUC​UAG​UCA​A-3′	S-protein			
5′-CGC​ACA​UUG​CUA​ACU​AAG​A-3′	Helicase			
5′-UUU​GUA​CUG​GUC​AAU​AUG​CUU-3′	S-protein	Aptamer-siRNA-LNP conjugate reduced the viral load by 50% *in vitro* and in a patient	Liposomes were used for delivery	Saify Nabiabad et al. (2022)

*In silico* design of siRNA.

**FIGURE 2:**
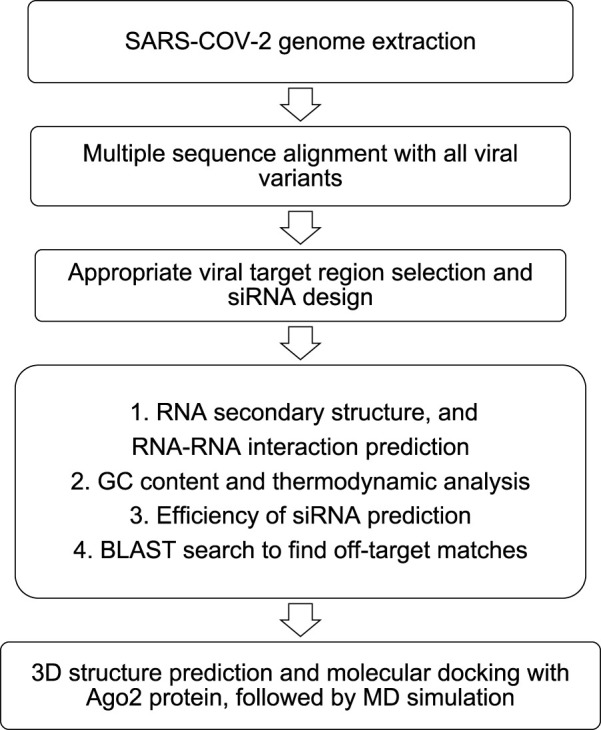
Computational pipeline to predict siRNA against target segments of SARS-CoV-2. Adapted with modifications from Ref. (Chowdhury et al., 2021).

The siRNAs are designed usually against the conserved region of the viral genome targeting the mRNA sequence responsible for the formation of the structural proteins (S, M, E, N), which help the viral particle to assemble and impart infectivity. To design well-targeted and precise siRNAs, computational approaches are followed with a preliminary retrieval of the genomes of the SARS-CoV-2 virus and variants. Conserved genomic regions across different variants are obtained by multiple sequence alignment with Clustal omega (Sievers and Higgins, 2018). Typically, these conserved regions across the different variants are regarded as the potential siRNA target sites. Next, with the help of web servers, siRNAs are designed considering Ui-Tei (Ui-Tei, 2004), Amarzguioui (Amarzguioui and Prydz, 2004), and Reynolds (Reynolds et al., 2004) rules. Many *in silico* siRNA prediction studies use multiple web servers to predict siRNAs targeted towards the same mRNA sequence and finally consider the common predicted siRNAs (Ayyagari, 2022). This approach ensures stringent shortlisting and robust applicability (Ayyagari, 2022). A few of the web servers include OligoWalk (Mathews and Sioud, 2010), i-Score Designer (Ichihara et al., 2007), siDirect v2.0 (Naito et al., 2009), and RNAxs (Tafer et al., 2008). To counter verify the thermodynamic suitability and readiness of the siRNAs, additional parameters are evaluated. The free energy of folding of the siRNA guide strand, along with the secondary structure prediction is performed using MaxExpect (Lu et al., 2009), DuplexFold, AccessFold (DiChiacchio et al., 2016), and ViennaRNA (Gruber et al., 2015) web-servers to rule out any RNA-RNA self-hybridization. Moreover, the efficiency of inhibition by the siRNA is predicted using SMEpred, siRNAPred, and VIRsiRNApred (Qureshi et al., 2013; Dar et al., 2016b). Further, heat capacity (C_P_)/melting temperature (T_m_) and GC content of the siRNA are predicted using DINA melt server (Markham and Zuker, 2005) and OligoCalc (Kibbe, 2007), respectively. Moreover, BLAST^®^ (Basic Local Alignment Search Tool) search against human genome is performed to identify off-target matches of the siRNA. Finally, 3D structure of the siRNA is predicted and computationally docked with Ago2 (PDB: 4OLA) followed by molecular dynamics simulation. Figure 2 summarizes the overall computational strategy to predict siRNA against target segments of SARS-CoV-2.

## Nanoparticles (NPs) used for the siRNA therapy of COVID-19

**FIGURE 3:**
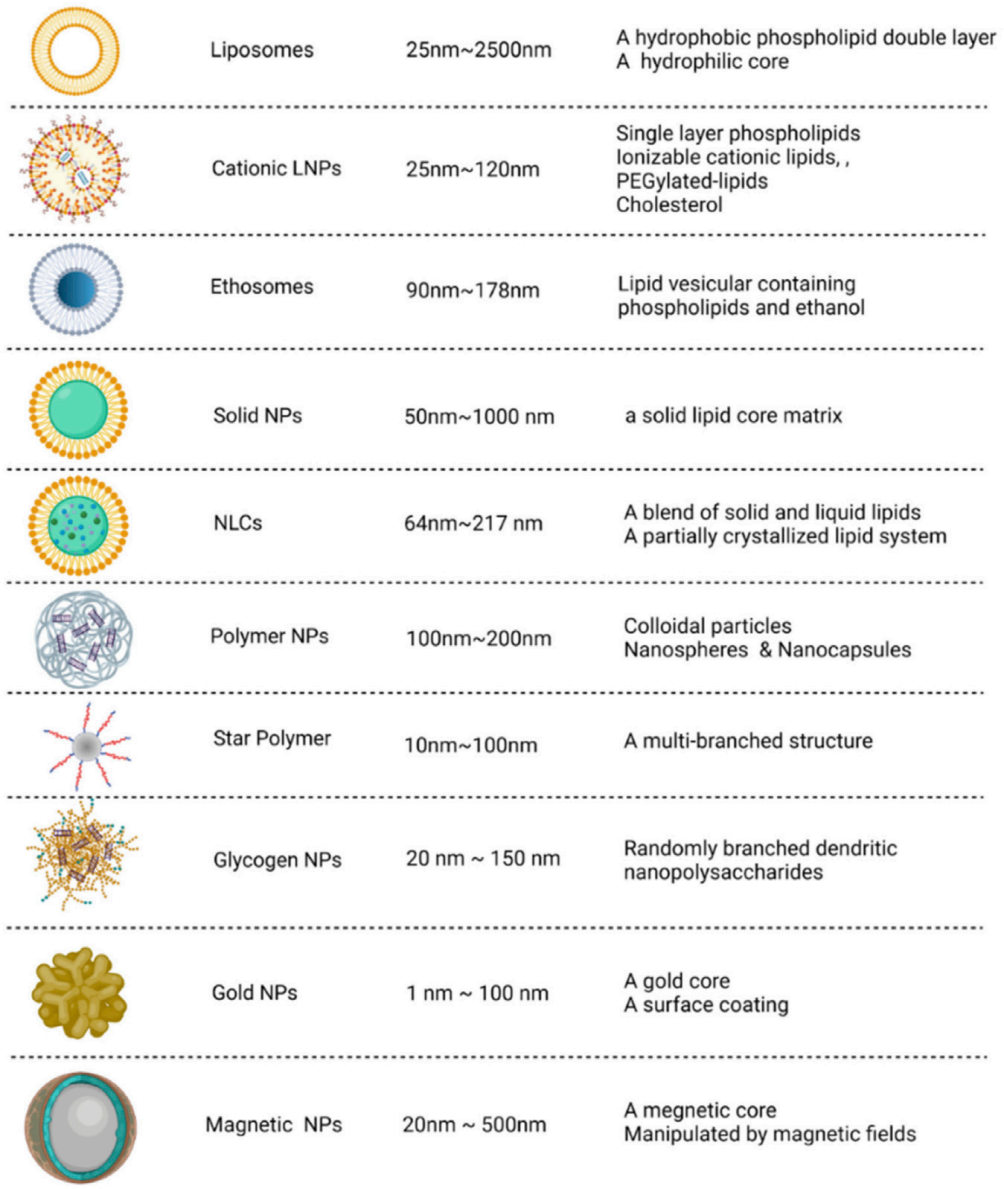
Potential nanocarrier configurations for siRNA delivery. The carriers can be categorized into organic (i.e., derived from lipid, polymeric and polysaccharides) and inorganic types. The size and structural features of these nanocarriers are depicted. Adapted from Ref. (Zhang et al., 2022a).

The administration of naked siRNA for *in vivo* application is challenging due to various biological barriers such as degradation by RNAases, instability of the molecule and the immune response that could neutralize the siRNA and can cause other adverse effects (Kalita et al., 2022). The carriers provide protection to the siRNA against biological factors and facilitate targeted delivery (Zhang et al., 2022a). For the treatment of COVID-19, the vaccines, Pfizer-BioNTech (BNT162b2) and Moderna (mRNA-1273) use lipid nanoparticles as carrier molecules. There are a number of suitable carrier configurations for the siRNA-based therapy as shown in Figure 3. Some of the potential nanocarriers are briefly discussed in this section.

### Lipid nanoparticles

**FIGURE 4:**
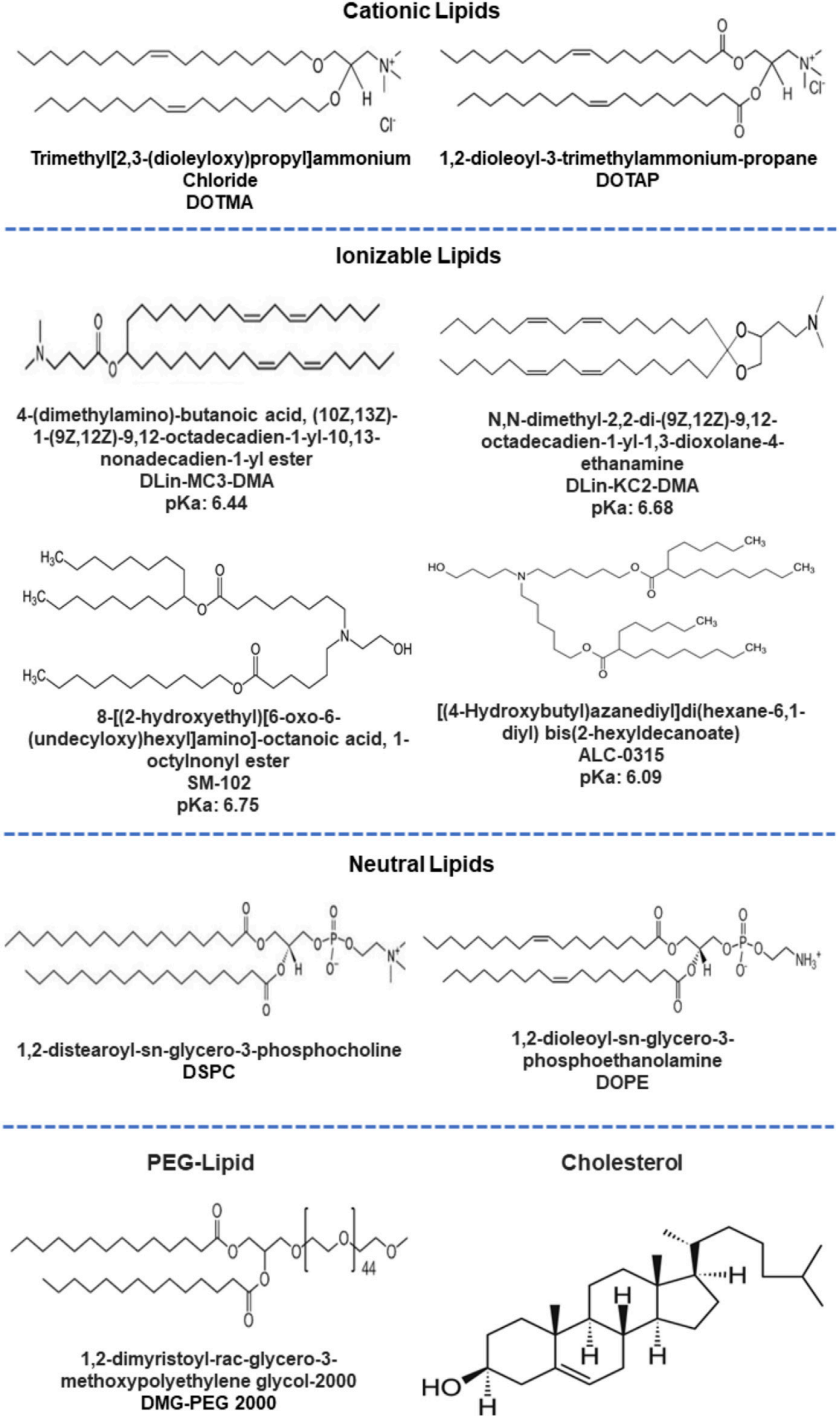
Different constituents (ILs, helper lipids, PEGylated lipids and cholesterol) used for lipid nanoformulations.

The lipid composition of the cellular membrane bilayer is the basis for designing and formulating the LNPs. The favorable interactions of the LNPs with the cell membrane determines the internalization, release, and stability of the payload (Bunea et al., 2020; Lee et al., 2022). Currently, LNPs are a popular strategy being explored for various diseases and applications (Chernikov et al., 2019), mostly attributable to the success of the mRNA-based vaccines against COVID-19. The LNPs are mainly composed of four components: Cationic or ionizable lipid (IL), cholesterol, helper lipid, and PEGylated lipid (Ly et al., 2022). Figure 4 represents the key constituents of LNPs. The cationic or ILs (also known as pH-sensitive lipids) interact with RNA molecules through electrostatic interaction between amine and phosphate groups. The interactions mediate the cellular internalization of nanoparticles along with siRNA and their release in the cytoplasm (Jayaraman et al., 2012; Lee et al., 2022). DLin-MC3-DMA [(6Z,9Z,28Z,31Z)-heptatriacont-6,9,28,31-tetraene-19-yl 4-(dimethylamino) butanoate], ALC-0315 {[(4-hydroxybutyl) azanediyl]di(hexane-6,1-diyl) bis(2-hexyldecanoate)}, and SM-102 {1-Octylnonyl 8-[(2-hydroxyethyl) (6-oxo-6 (undecyloxy)hexyl)amino]-octanoate} are clinically approved lipids, among which ALC-0315 and SM-102 have been applied for mRNA delivery, and DLin-MC3-DMA (MC3) has been used for siRNA delivery for treating transthyretin amyloidosis (Suzuki and Ishihara, 2021; Urits et al., 2021; Saadati et al., 2022). The helper lipids are generally phospholipids such as DOPE (dioleoylphosphatidylethanolamine) and DSPC (distearoylphosphatidylcholine) and are used for the stability of LNPs and for aiding the endosomal release (Hou et al., 2021). DSPC has been used in the approved vaccines, improving structural stability through the formation of the lamellar phase while DOPE is proven for endosomal disability and release (Koltover et al., 1998). Cholesterols are responsible for structural integrity and promotes membrane fusion (Cheng and Lee, 2016). The PEG moieties enhance the colloidal stability and prevent the aggregation of serum protein on the LNPs surface and immune response (Suk et al., 2016; Sebastiani et al., 2021).

Along with the conventional composition, Bogaert *et al.* have attempted the repurposing of cationic amphiphilic drugs (tricyclic antidepressants and antihistamines) for formation of cationic lipid vesicles for mRNA delivery. These drug molecules, due to their amphiphilic properties, accumulate in acidic lysosomes in their active form. The formed complex can be used to co-deliver mRNA within cationic amphiphilic drugs-assisted LNPs for various applications (Bogaert et al., 2022). The approved LNP-based therapeutic formulations such as Patisiran (Alnylam), Elasomeran (Moderna) use similar ratios (50:10:38.5:1.5) of ILs, helper lipid (DSPC), cholesterol and polyethylene glycol lipids (Suzuki and Ishihara, 2021; Ferraresso et al., 2022). The said molar ratio has been reported by many studies as optimum and highly potent (Carrasco et al., 2021; Suzuki et al., 2022). Pfizer-BioNTech mRNA vaccine Tozinameran is based on a lipid molar ratio of 46.3:9.4:42.7:1.6 (ALC-0315:DSPC:Cholesterol:ALC-0159) (Schoenmaker et al., 2021).

**FIGURE 5:**
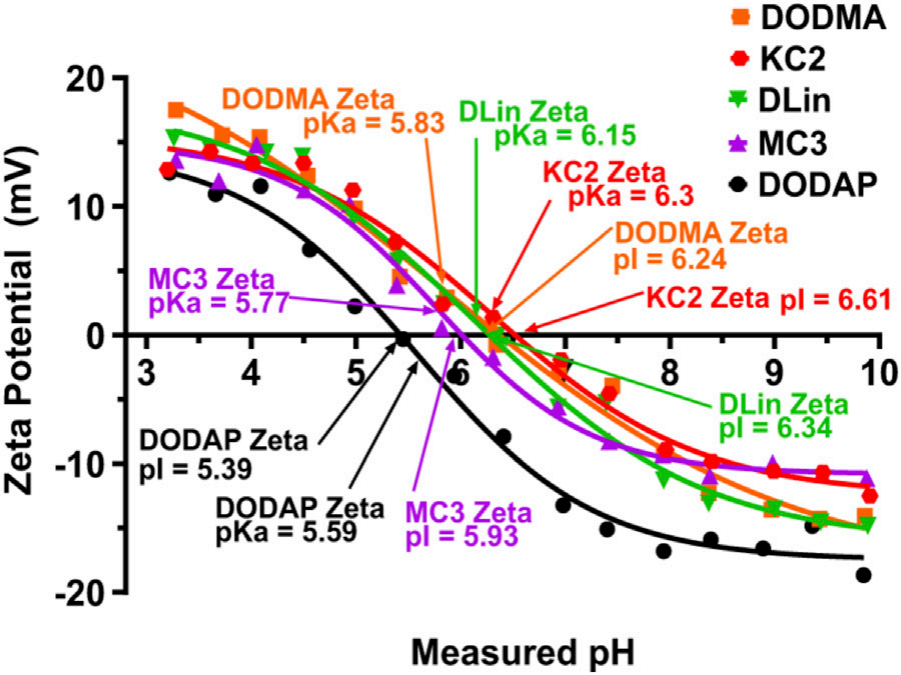
The ionization characteristics of the commercially available ILs obtained through zeta potential measurement. The LNPs showed a transition of charges-positive zeta potential at low pH and negative zeta potential at high pH, covering the range of endosomal and lysosomal pH. Adapted from Ref. (Carrasco et al., 2021).

*Properties of LNPs.* The selection of ILs or pH-sensitive lipids depends on the acid-dissociation constant (pKa) value. These pH-sensitive lipids possess deprotonated tertiary amine head groups at the physiological pH, but acquire positive charges at pH below pKa. Thus, the ILs interact with the negatively charged siRNA molecules through electrostatic interactions enabling neutralization. The neutral surface charge under the physiological conditions of blood and serum eases the LNP internalization through the plasma membrane. The protonation of the head groups in the acidic condition in the cytoplasm helps destabilize the LNPs structure to release siRNA molecules (Zhi et al., 2013; Albertsen et al., 2022; Syama et al., 2022). The pKa value of the head group of ILs determines the surface charge, which ultimately affects biodistribution, cellular internalization, and endosomal release. Studies have reported that lipids with pKa values between 6 and 6.6 showed well *in vivo* activity (Rajappan et al., 2020). Carrasco *et al.* studied the pKa values of some commercially available lipids and stated that the lipids with pKa values between 6 and 7 are optimum for RNA-based therapeutics, considering the endosomal release at acidic conditions. The variations in the zeta potential of nanoparticles derived from commercial lipids and the pKa values of the lipids are as shown in Figure 5 (Carrasco et al., 2021). The ionizable lipid DLin-MC3-DMA with an apparent pKa 6.44 used in Onpattro^®^ was identified from a library of 56 ILs consisting of a dilinoleyl-based hydrophobic tail with varying headgroups (Jayaraman et al., 2012).

The tail length of the lipids determines the fluidity in the bilayer *via* the carbon length and the structure of the aliphatic chain (Zhi et al., 2010). The linker between the head group and tail affects the stability, cytotoxicity, and other aspects. The internalization and the release of the nucleic acid molecules are majorly dependent on the linker properties and thus the linker plays a major design role for the performance of LNPs (Zhi et al., 2018). Based on the linking bonds present, the lipids can be categorized into ether, ester, disulfide, phosphate, and other types. The approved cationic lipids, namely, DLin-MC3-DMA, ALC-0315, and SM-102, have bio-cleavable ester linkers which help dissociate the lipid-siRNA complex and thus the release of siRNA (Maier et al., 2013). The degraded fragments of the lipids are rapidly cleared from the body allowing for multiple doses within a short duration.

Lipidoids. Lipidoids are a novel class of lipid-like molecules resembling cationic lipids with alkylated tetraamine backbone. The clinically approved siRNA-based drug, Onpattro, is indeed based on lipidoids. The chemically synthesized lipidoids exhibit an extensive library of over 1,200 diverse lipids in one study with the potential in siRNA delivery for specific gene silencing purposes owing to pKa values between 6 and 7 (Dormenval et al., 2019). Khare *et al.* have explored the potential of lipidoid C12-200 in the formulation of a delivery vehicle owing to excellent knockdown efficacy and cellular uptake (Khare et al., 2022). The prepared lipidoid CS12-200 nanoparticles showed 83.8% siRNA loading when formulated with or without PEGylated helper lipids. The transfection efficacy for siRNA on neural cells was also increased twice without toxicity. Thus, the suitability of lipidoids for the delivery of siRNA for various purposes can be explored.

### Liposomes

**FIGURE 6:**
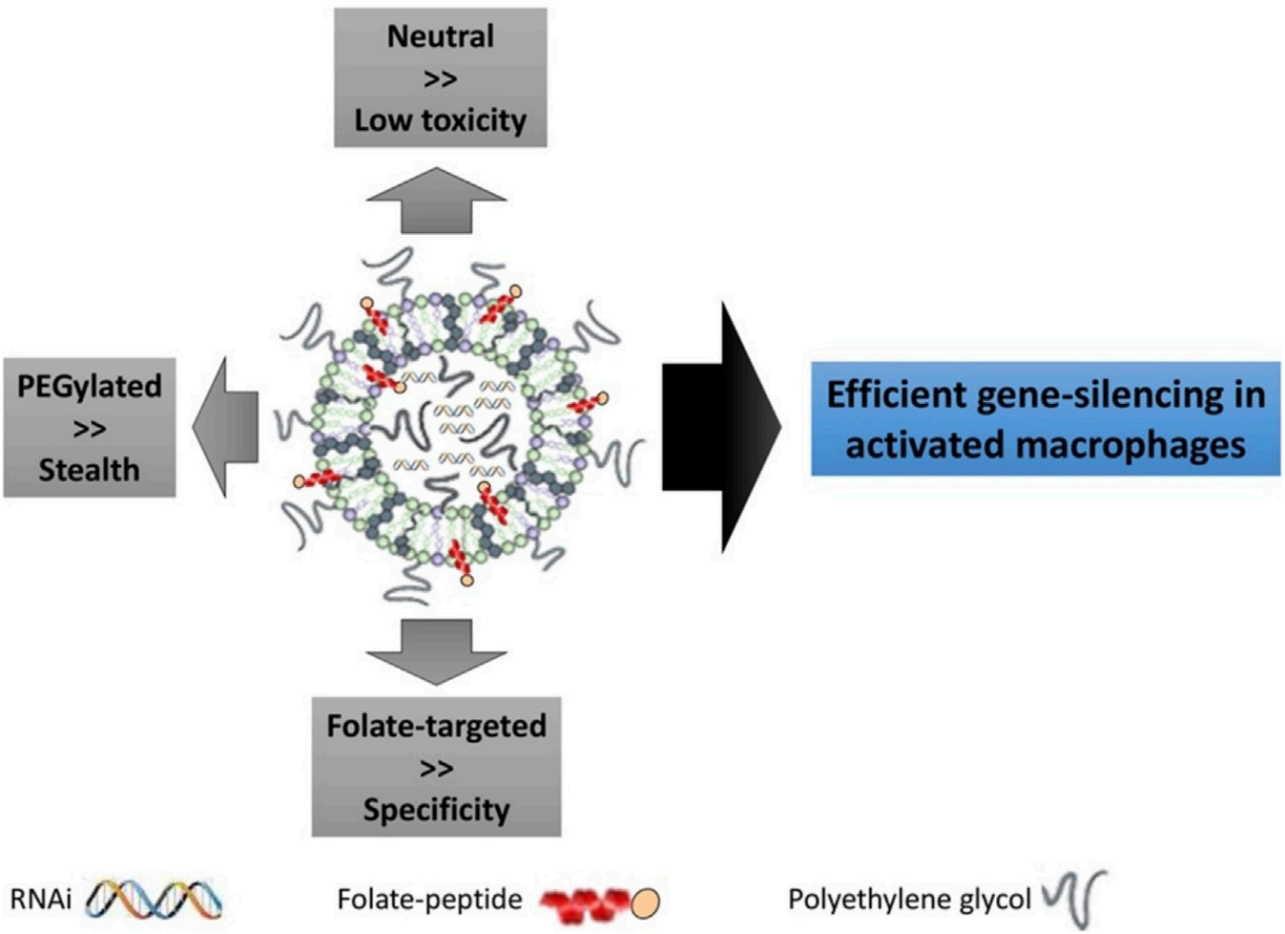
Folate incorporated liposome formulation for gene silencing applications. siRNA for Mcl-1 (a protein expressed in the rheumatoid joint macrophages) silencing was loaded in liposome and targeted towards folate receptor. PEGylated liposome offered stealth features against phagocytic uptake; neutral lipids exhibited low toxicity while specific targeting was observed by anchoring folate-targeted peptides. Adapted from Ref. (Nogueira et al., 2017).

Liposomes are spherical vesicles composed of phospholipid bilayers with an aqueous core. These structures are advantageous due to their capability of carrying both hydrophilic siRNA molecules within the core and lipophilic drugs within the bilayers (Chadar et al., 2021). Liposomes can be prepared using mechanical dispersion methods such as ultrasonication, membrane extrusion, thin film hydration, and microfluidics (Al-Amin et al., 2020; Has and Sunthar, 2020; Jo et al., 2020; Zhang and Sun, 2021). Liposomes with sizes 100–1,000 nm are referred to as large unilamellar vesicles, while small unilamellar vesicle sizes range from 20–100 nm in size (Dymek and Sikora, 2022). Similar to all nanoparticles, the size and charge of liposomes determine the blood circulation time and cellular uptake rates (Ren et al., 2019; Lee et al., 2022). Nogueira *et al.* showed efficient siRNA delivery to activated macrophages using neutral lipid DOPE-based PEGylated liposomes (Nogueira et al., 2017), as shown in Figure 6. The DOPE-based liposomes with PEG exhibited almost neutral surface charge and thus showed a higher stealth degree, i.e. reducing the uptake by mononuclear phagocyte system. Further, the PEGylated liposomes anchored with folate targeted peptides showed high specific delivery of siRNA for gene silencing applications. In another aspect, the cationic liposomes showed more internalization efficiency than anionic liposomes due to their better interactions with the negatively charged plasma membrane. However, cationic liposomes may generate reactive oxygen species causing cytotoxicity (Kulkarni et al., 2018). Lechanteur *et al.* concluded that the cytotoxicity by cationic liposomes complexed with siRNA was dependent on the molar ratio of nitrogen on the IL to phosphate on RNA (N/P) and they can be safely used with the N/P ratio of 2.5 (Lechanteur et al., 2018).

### Polymer nanoparticles

The application of polymers for siRNA delivery has been well explored. Common biodegradable polymers such as poly (lactic acid) (PLA), poly (glycolic acid) (PGA), and poly (lactic-co-glycolide) (PLGA) are already approved by the Food and Drug Administration (FDA) for drug delivery applications. These polymers offer excellent biocompatibility and low immunogenicity (Wood, 2018; Saeed et al., 2021). Similar to lipids, surface charges of the resultant nanoparticles and the molecular weight of the constituting polymer influence siRNA delivery to the desired site. Cationic polymers such as polyethyleneimine and poly (l-lysine) have also been explored. However, these polymers show increased toxicity with increasing molecular weight. Karimov *et al.* have attempted the small linear polyethyleneimines modified with tyrosine for enhanced siRNA attachments (Karimov et al., 2021). The γ-[32P]-ATP labeled siRNA was efficiently transfected in three different xerographs without exhibiting toxicity. The knockdown efficacy in H441-luc cells due to the polymer-tyrosine-siRNA complex was also found efficient, proving the capabilities of polymeric nanoparticles for siRNA delivery.

Further, studies have reported using low molecular weight polymer polyethyleneimine (PEI) successfully for siRNA carriers against breast cancer (Aliabadi et al., 2020; Uludağ et al., 2020). Also, the lipophilic PEIs was used for carrying the siRNA for the toxicity studies on human lung fibroblast cells and delivery of siRNA against Human Coronavirus 229E. The polymer siRNA complex showed more than 85% cell viability, and their transfection efficiency was similar to reference Lipofectamine™ (Montazeri Aliabadi et al., 2021). Further, biodegradable cationic polymers with ester bonds, such as poly (beta-amino ester), are used for siRNA delivery as they offer effective endosomal escape, flexible conjugate binding capabilities, high stability, and tunable charge density (Nezhad, 2022).

## Preparation of small interfering RNA-loaded lipid nanoparticles

**FIGURE 7:**
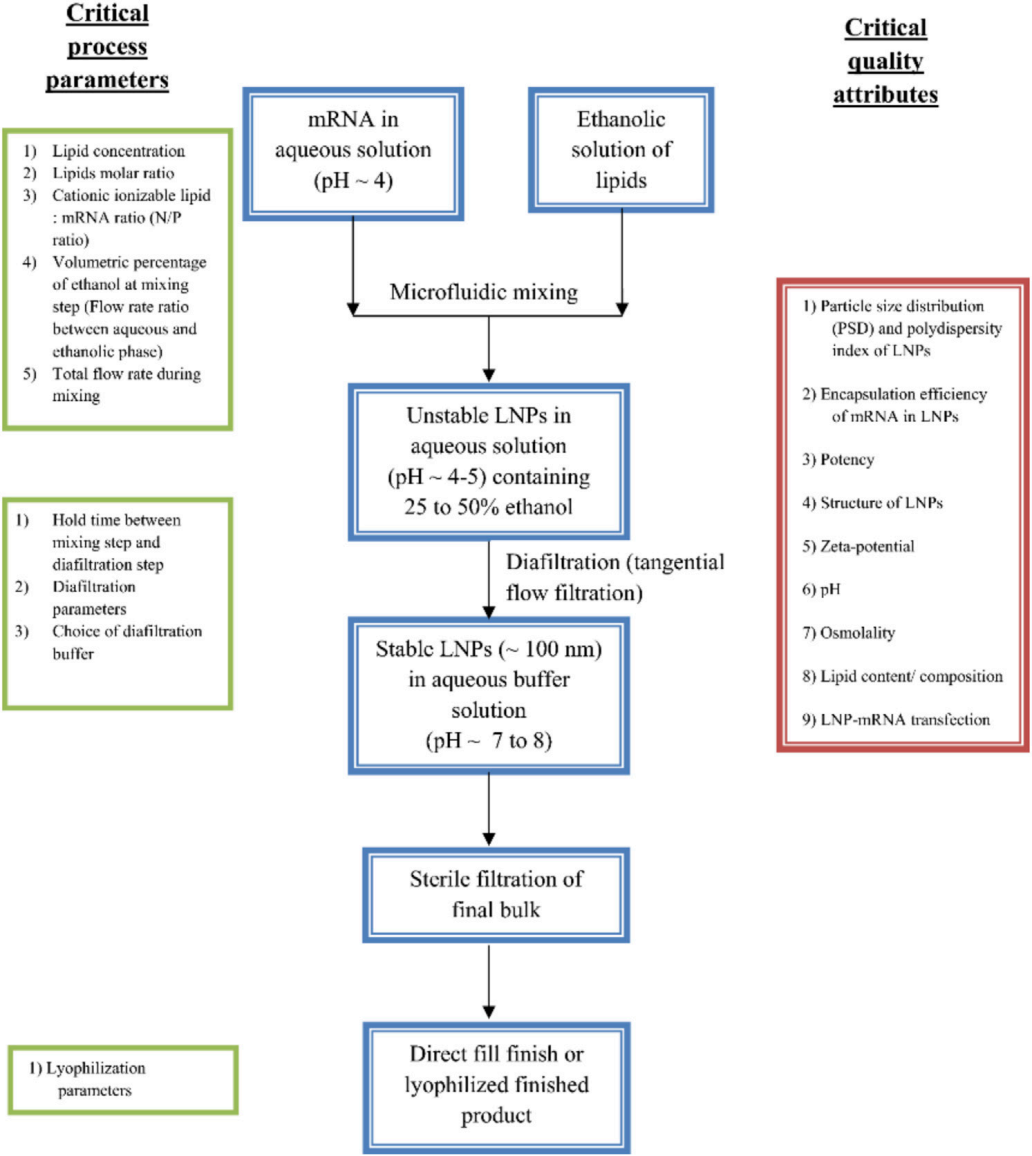
Microfluidics method for preparation of LNPs formulations. The preparation methods can be optimized through various process parameters such as ratio of constituent lipids, molar ratio of ILs and RNA (N/P ratio), the mixing flow rate and time, holding time, filtration and storage conditions. After the preparation, the LNPs can assess for their performance through qualitative analysis through size, charge and transfection and loading efficiency [adapted from (Ramachandran et al., 2022)].

The conventional preparation method for lipid nanoparticles, called thin film hydration, includes the dissolution of the constituent lipids in organic solvents followed by the gradual evaporation of the solvent to form a thin layer of dried lipids. The film is then rehydrated with a buffer containing the carrier molecules such as RNA or drugs (Zhang et al., 2021a). Another popular method for LNPs preparation includes the dissolution of a mixture of constituent lipids in ethanol and mixing it with the aqueous phase containing the load molecules such as siRNA (in phosphate buffers). The homogeneity of the mixing in such methods was inadequate leading to higher polydispersity index. With the advancement in instrumentation, microfluidic chambers mix these two solutions at a particular flow rate to obtain the desired size range of LNPs (Ly et al., 2022; Masatoshi et al., 2022; Younis et al., 2022). The obtained LNPs solution is further concentrated using dialysis and quantified for the extent of loading for nucleic acid molecules. Alternatively, polycarbonate-based membrane filters can prepare narrow-sized LNPs (Syama et al., 2022). Figure 7 depicts the steps involved in the preparation of LNPs using microfluidic method. The synthesis methods should critically consider some process parameters such as the molar ratio of lipid components, N/P ratio, and the flow rate of mixing (in the case of microfluidic chambers). The prepared LNPs can be qualitatively characterized through particle size distribution, structure of LNPs (as shown in Figure 3), surface charges and pH for their stability performance. While, siRNA encapsulation and transfection efficiency determine the efficacy/potency of prepared LNPs as therapeutics. The detailed perspective on the preparation of LNPs for siRNA delivery can be found elsewhere (Aldosari et al., 2021; Tenchov et al., 2021).

## Stability of lipid nanoparticles

For effective therapeutic use of an LNP product, the formulation lipids should be rapidly metabolized *in vivo*, yet exhibit good chemical stability in order to maintain sufficient shelf-life and initial circulation time. During the circulation period in extracellular fluids and intracellularly, several factors such as ionic strength, pH, adsorbing proteins and other environmental conditions may destabilize the LNPs, which can considerably affect the LNPs efficiency (Shah et al., 2022). Hence, stability of lipids under various conditions is an important aspect on protect the gene payloads, enable efficient delivery into target cells and assure functional outcomes *in vivo* (Koitabashi et al., 2021; Kon et al., 2022).

**FIGURE 8:**
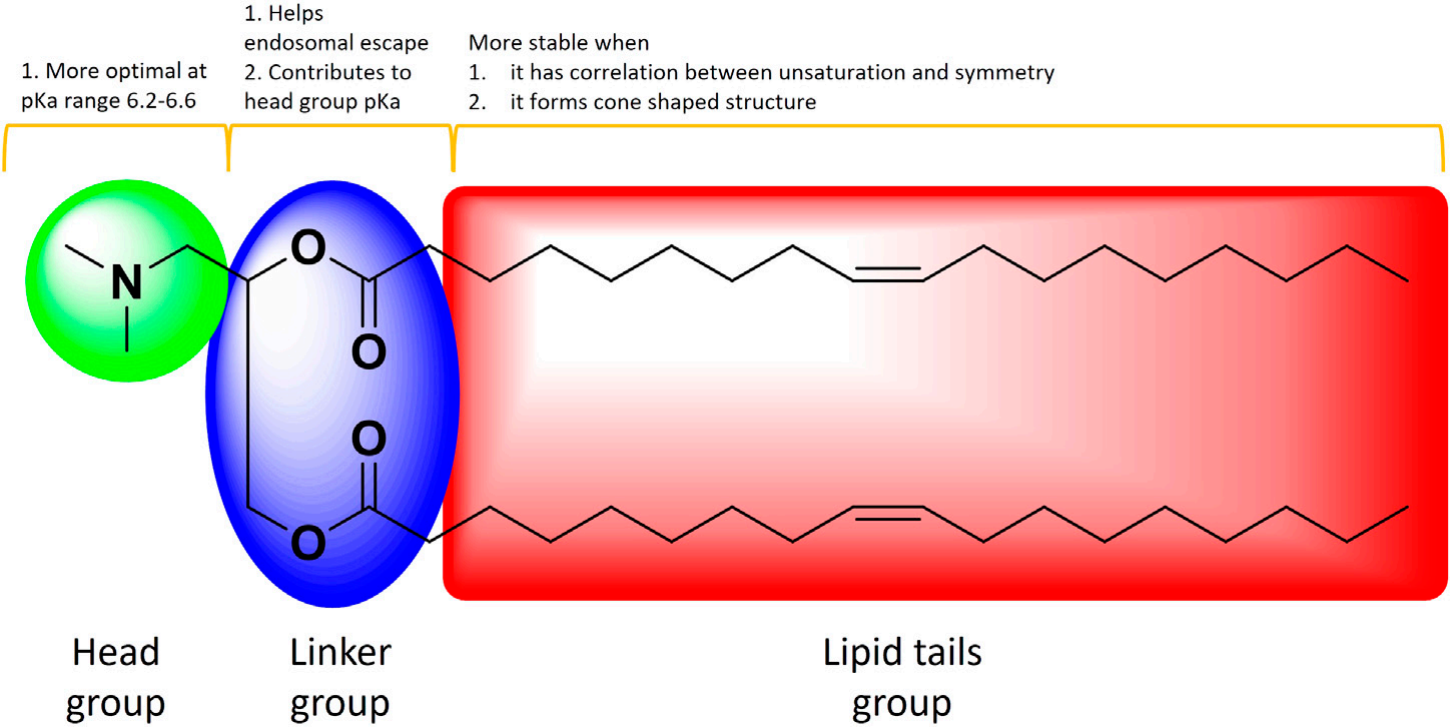
The schematic of an IL illustrating the main elements and how they contribute towards stability. Adapted from Ref (97)

The ILs (Figure 8) are inspected frequently to find the ideal one for efficacy while maintaining muted toxicity profiles (Paramasivam et al., 2022). The elements of ILs, the ionizable head groups, linkers and the hydrocarbon tail chains, offer significant advantages in stability features (Albertsen et al., 2022). The mRNA-1273 LNP vaccine with ionizable amino-alcohol head group has pKa 6.75; the pKa range 6.2–6.6 was suggested to be optimal for protein expression following IV delivery, which has been consistent with effective mRNA-1273 use (Hassett et al., 2019). The linker between ionizable head group and the hydrocarbon chain contributes to head group pKa and LNPs endosomal escape potential (Maier et al., 2013). The hydrocarbon chain tails help LNPs by altering the endosomal escape, stability during storage and toxicity (Suzuki et al., 2017). The hydrophobic tails with unsaturation and symmetry contribute to LNP stability. For instance, branched hydrophobic chains can be advantageous in the context of endosomal escape by creating a cone shaped structure (Zhang et al., 2021b).

**FIGURE 9:**
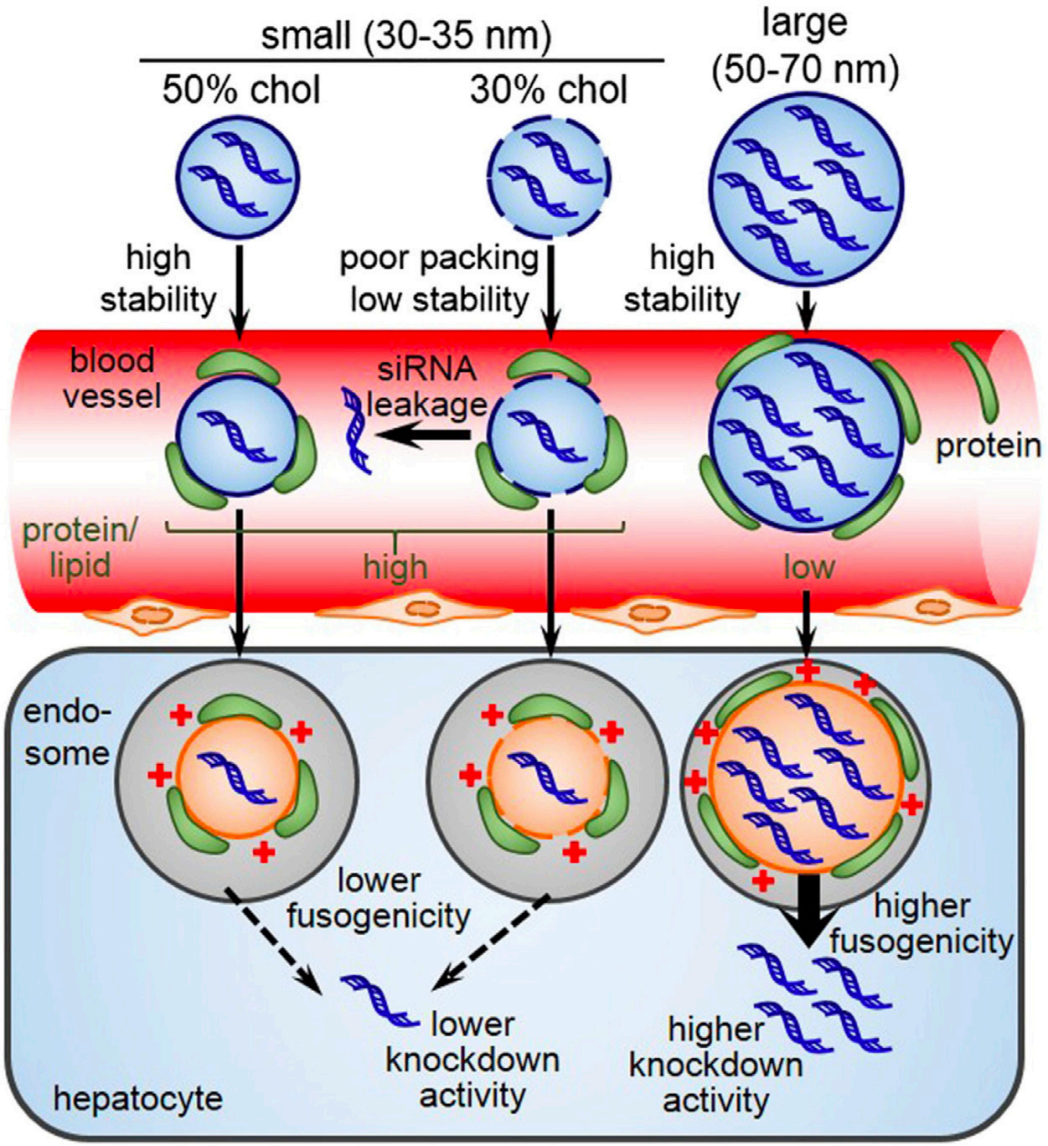
Schematic illustration of the stability of LNPs based on size. Adapted from Ref. (Sato et al., 2016).

Size of the LNP is another important factor that plays a vital role in stability as well as pharmacokinetics. A study found that small-sized LNPs (<35 nm) showed a tremendous down in lipid packing, stability, ability for endosomal escape, and the cause appears to a higher amount protein being adsorbed on the surface of LNPs (Sato et al., 2016) (Figure 9). Cabral et al., found only <50 nm nanoparticles can penetrate poorly permeable hypovascular tumors. Furthermore, increasing the permeability of hypovascular tumors using TGF-β signaling inhibitor improved the accumulation of >70 nm micelles, offering a way to enhance the efficacy of larger nanomedicines (Cabral et al., 2015). The stability of small LNPs in the blood circulation was increased by cholesterol a known helper lipid which increases the packing of lipids with unsaturated chains and therefore stabilize LNPs and avoid siRNA leakage (Hung et al., 2007).

## Cleavage of lipid nanoparticles

**FIGURE 10:**
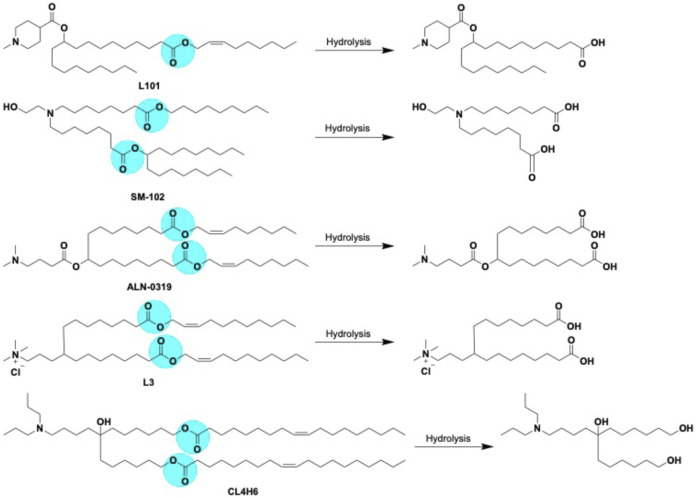
Biodegradable LNPs L101, SM-102, ALN-0319 (adapted from ref 113) L3 (adapted from ref 112) CL4H6 (adapted from ref 111) with cleavable ester linkage sites (highlighted in blue color) and expected hydrolysis pathway catalyzed by esterases.

The prime goal of developing degradable ILs is to improve lipid metabolism and prevent associated toxicities. The pharmacokinetic properties of ILs were strongly improved by introducing easily cleavable ester linkages into the tail hydrocarbon chain (Prata et al., 2004). Ester bonds is chemically stable under physiological conditions, but can be hydrolyzed by endogenous esterase or lipase in tissues and intracellular compartments (Luten et al., 2008). A summary of the hydrolytic cleavage pathway of lipids is shown in Figure 10. One or more ester linkages in the hydrophobic tail as well as the linker region appeared to be particularly interesting *in vivo* because of their on-demand degradation feature by esterases, which mitigate the pharmacokinetic properties of lipids with negligible toxicity (Sabnis et al., 2018). Owing to its ability to alter the head group pKa, the LNPs with ester linkage near to the head group suppresses the efficacy. In contrast, placing the ester linkage near the terminal ends of the lipid tail had little effect on the head group pKa and did not alter the *in vivo* outcome of the corresponding LNPs. LNPs formulated with the incorporation of ester linkages into the hydrocarbon chain region of the amino lipid preserved the head group linker structure with demonstrated efficacy (Gilham and Lehner, 2005). One study explored fully biodegradable ester bonds in the hydrophobic tails; the hydrolysis produced water-soluble alkanol amine that were readily eliminated from tissues, resulting in a reduced toxicity and liberation of endogenous oleic acids (Sato et al., 2019). Alternatively, replacing double bonds with ester linkages produces hydrolytic cleavage products that are quickly merged into catabolic pathways, without dropping capability. Multiple studies have shown such biodegradable ILs containing ester bonds attained instant elimination and excretion as well as significant permissibility in rodents and non-human primates after intravenous (Sato et al., 2019) and intramuscular (Hassett et al., 2019) administration. The fragmented lipids need to be metabolized in the plasma because these smaller fragments are often carried into systemic circulation. Hence, plasma stability is an applicable measure of overall biodegradability and potential for accumulation over time (Pei et al., 2022).

## Targeted delivery of nano-formulations

The targeting of LNPs is crucial for the success of the treatment process. The conventional LNPs resemble the low-density lipids and thus can be adsorbed by Apolipoprotein E (ApoE) in the blood. These adsorbed LNPs typically accumulate in the liver and their hepatocytic uptake occurs *via* various lipoprotein receptors (Tian et al., 2019; Younis et al., 2022). Thus, targeting organs or tissues other than the liver is complex and inefficient by the conventional LNPs (Morán et al., 2022). Better understanding and control of the LNP fate *in vivo* is important and this has been actively explored in numerous studies.

### Ionizable cationic lipids

Algarni *et al.* have explored the targeting efficiency of three ionizable cationic lipids, DLin-MC3-DMA, DLin-KC2-DMA and DODAP (1,2-dioleoyl-3-dimethylammonium-propane), for the organ-specific delivery of pDNA (Algarni et al., 2022). The intravenous administration in mice models with LNPs formulations showed that the DLin-MC3-DMA and DLin-KC2-DMA bearing LNPs more precisely and efficiently transfected the nucleic acid cargo to the spleen instead of the liver. The structure of ILs, DLin-MC3-DMA and DLin-KC2-DMA with two double bonds per alkyl chains, has influenced the transfection efficiency in the spleen. Thus, the selection of suitable ILs may improve the targeting.

### Aptamer

**FIGURE 11:**
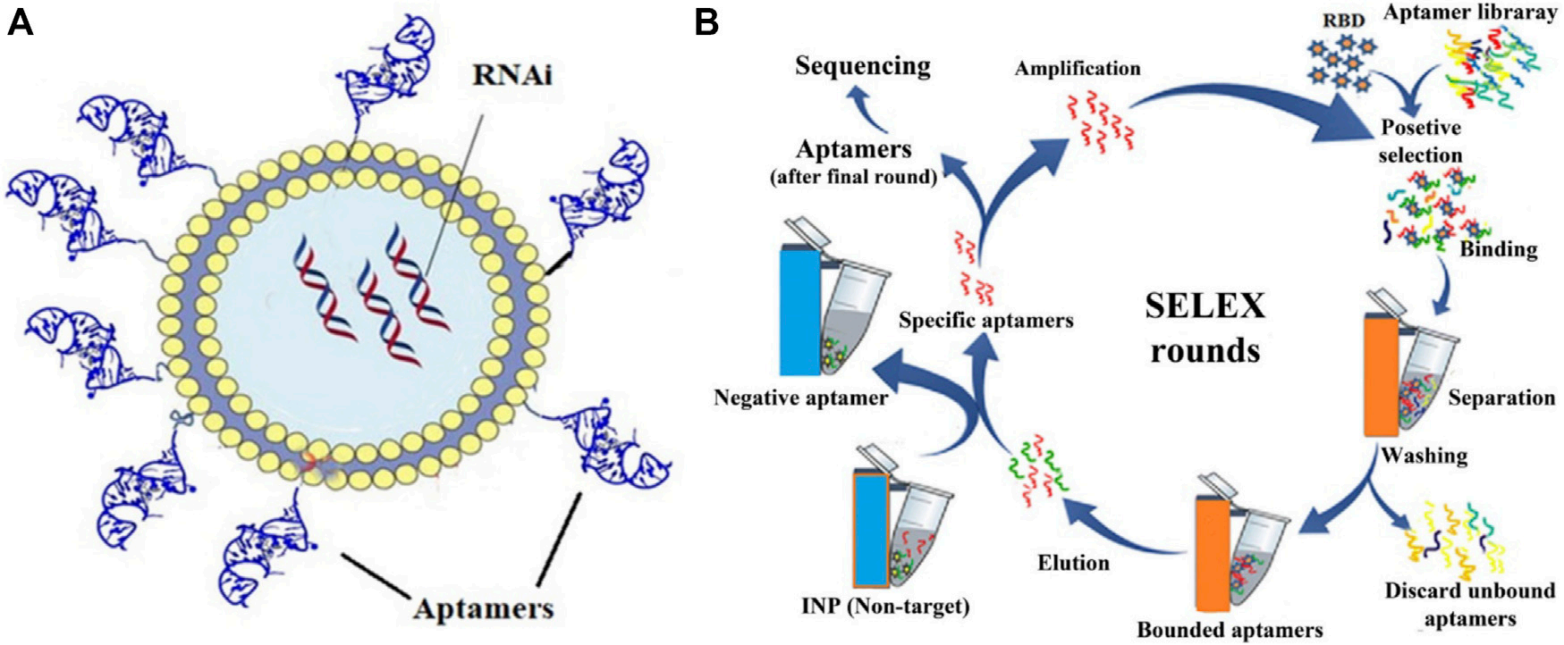
**(A)** The structure of aptamer molecules conjugated onto LNP-RNAi complex. LNPs were first complexed with siRNAs, which were then conjugated with synthesized aptamers. **(B)** SELEX-based method for aptamer selection after multiple rounds of binding, separation, washing, elution and amplification. Adapted from ref. (Saify Nabiabad et al., 2022).

Besides the difficulty of the naked siRNA passing through the cell membrane, pulmonary proteases, mucus layer, and macrophage-related inflammation also pose obstacles to siRNA delivery (Terada et al., 2021). Organ and site-directed targeting of siRNAs is also a significant challenge in the delivery and aptamers, which are highly specific single-stranded oligonucleotides directed against a target, could help in this approach (Khanali et al., 2021). For use in COVID-19 disease, aptamers prepared against the receptor binding domain (RBD) of the viral spike protein were prepared by the SELEX (Systemic Evolution of Ligands by EXponential enrichment) method and then conjugated with the siRNA bearing LNP conjugates (Saify Nabiabad et al., 2022) as shown in Figure 11. A study used 50–90 µM of aptamers to siRNA-LNP conjugate containing around 40–80 nM of siRNA and found ∼50% inhibitory reduction *in vitro* in SARS-CoV-2 copies. This study also included a case study of a SARS-CoV-2 patient administered 10 mg of the aptamer-siRNA-LNP formulation by inhalation for 6 days, which indicated improvement in overall conditions as indicated by chest radiological and biochemical observations (Saify Nabiabad et al., 2022). The LNP contained DMKE (45%) (O,O′-dimyristyl-N-lysyl glutamate), DSPE-PEG2000 (4%), and cholesterol (46%), and was prepared by a methanol and chloroform mixture (2:1, v/v).

Many aptamers have been additionally developed for blocking the interaction of the S protein-ACE2 receptor, preventing viral entry (Gupta et al., 2021a; Li et al., 2021; Schmitz et al., 2021). Considering the high mutation rate of the spike protein, the search for a universally developed aptamer targeting all available variants is challenging. One such recent study designed an aptamer ‘MSA52′from a library of specifically curated aptamers with K_d_ values of 2–10 nM targeting the wildtype, and B.1.1.7 (Alpha), B.1.351 (Beta), P.1 (Gamma), B.1.429 (Epsilon), B.1.617.2 (Delta), B.1.1.529 (Omicron) variants (Zhang et al., 2022b). However, the *in vitro* and *in vivo* targeting aspects of these aptamers are yet to be verified.

### Selective organ targeting

**FIGURE 12:**
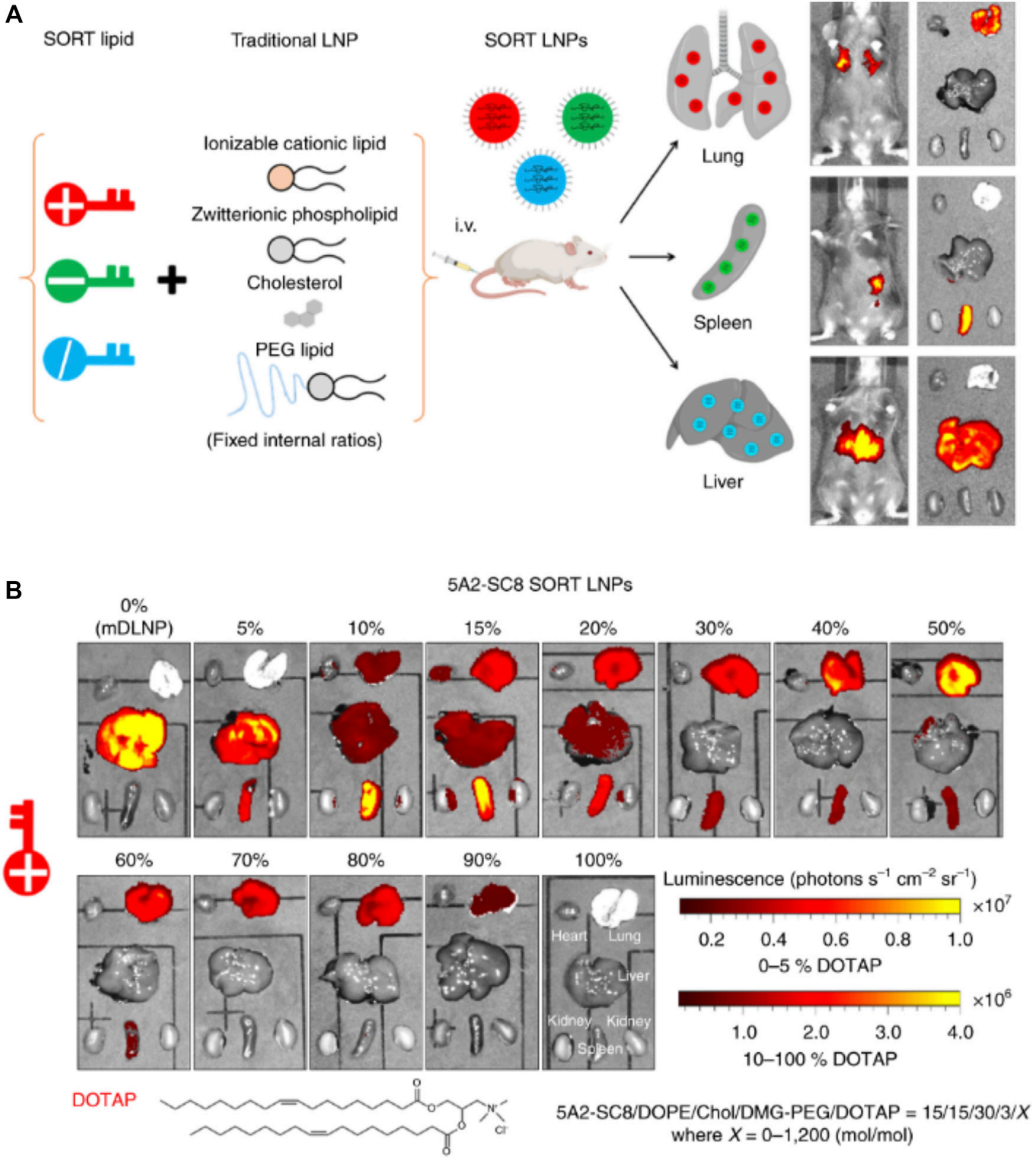
**(A)** Preparation of SORT LNPs for mRNA delivery for liver, spleen and lungs by incorporation of different SORT lipids in the formulation of LNPs. **(B)** The level of luciferase expression after administration of 5A2-SC8 SORT LNPs in mice models. With the increased cationic lipid DOTAP percentage, the expression of luciferase shifted from liver to spleen to lungs.

Selective organ targeting (SORT) is a new technique for the regulated delivery of siRNA to specific targets with the help of LNPs. In conventional LNPs nanocarrier systems, the balance of ionizable groups and hydrophobicity of lipid nanoparticles determines the effective intracellular delivery. Such nanocarriers, may not show efficient targeting for various organs, except for liver. However, the SORT approach uses the unbalanced charge of lipids to alter the tissue tropism through the functional groups present and physiochemical properties of SORT molecules (Cheng et al., 2020). The design of the SORT includes introducing a fifth component into the formulation of LNPs without destabilizing the actual structure. The added SORT molecule controls the biodistribution, apparent pKa, and interaction with serum protein. The change in tissue tropism depends on surface charges of SORT molecules and their amount, which may help to predict the targeting of the LNPs for expression of load in particular organs (Wang et al., 2022). Compared to other targeting strategies such as aptamers or antibodies, SORT molecules offer innate targeting without any surface modifications. During the circulation, PEG-lipids of SORT-LNPs get desorbed, exposing the SORT molecules, followed by the adsorption of distinct serum proteins. These protein-adsorbed LNPs interact with specific receptors expressed by cells of the target organ (Cheng et al., 2020; Dilliard et al., 2021). Cheng *et al.* used an engineered degradable dendrimer-based ionizable cationic lipid to target the lung, spleen, and liver. Here, the 5A2-SC8 SORT lipid fraction (0%–100%) was added to DOTAP. The shift in target from the liver to the spleen to the lungs was observed as an expression of luciferase protein, as shown in Figure 12. Another SORT molecule, 4A3-SC8, was used with 20% DODAP for liver targeting, 50% DOTAP for lung targeting, and 10% 18 PA for spleen targeting (Wang et al., 2022).

Effects of incorporation of SORT molecules in LNPs formulations visible from luminescence profile for tested mice models. Adapted from Ref. (Cheng et al., 2020).

## Release of small interfering RNA

**FIGURE 13:**
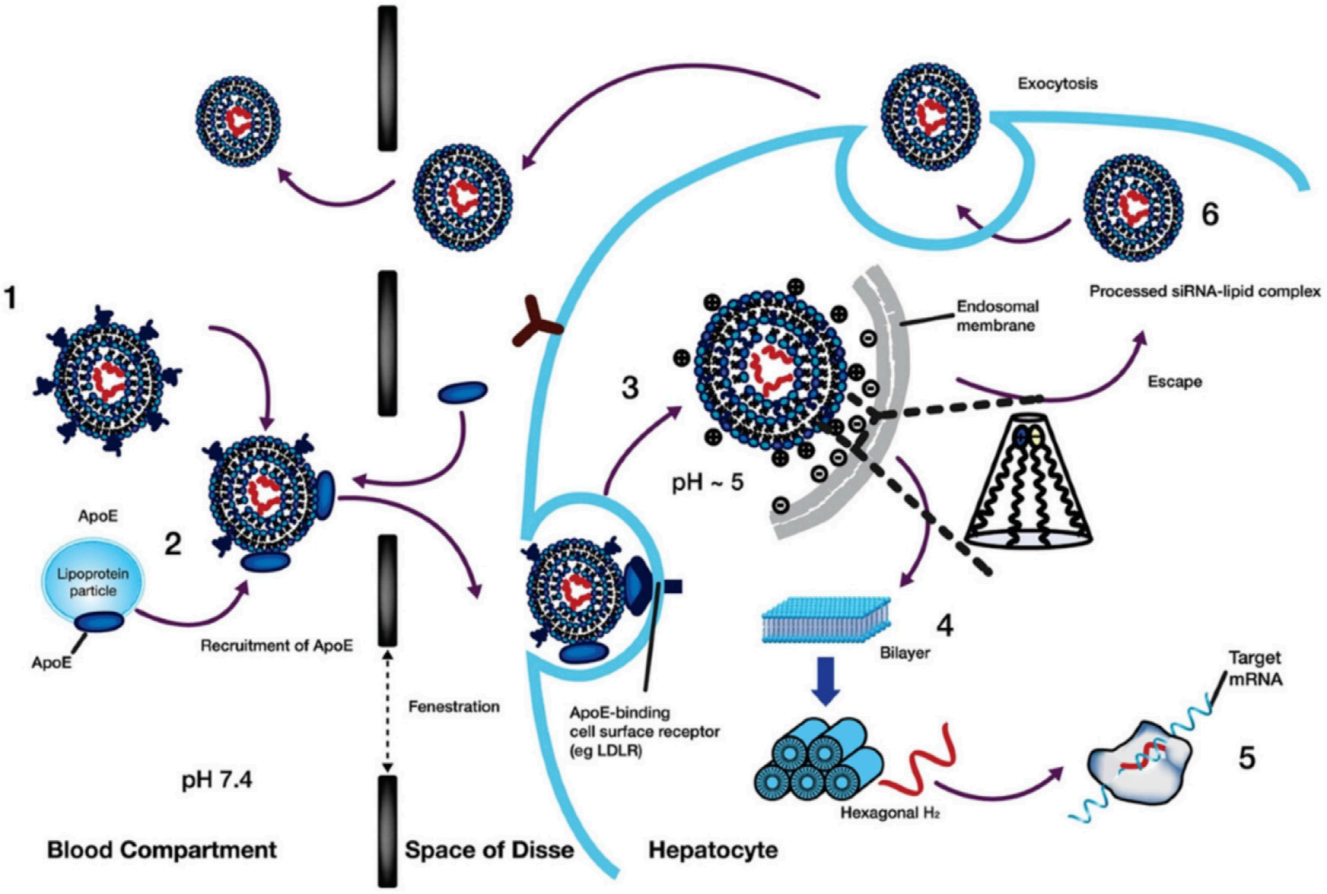
Internalization and release of RNA molecules in the hepatocytes. At physiological pH 7.4, the LNPs are adsorbed with ApoE protein which binds to low-density lipoprotein receptors in hepatic cells. The endocytic internalization of LNPs resulted in protonation of the surface due to acidic pH 5. The destabilization LNPs alters the hexagonal-like structure to release the loaded RNA molecules within the cell membrane for upregulation (mRNA) or downregulation (siRNA). The destabilized LNPs structure disintegrates and is removed while some LNPs face endosomal escape. Adapted from Ref. (Schlich et al., 2021).

The internalization of LNPs occurs through the endocytosis process (Lee et al., 2022). In the case of hepatocytes, the serum proteins such as ApoE are hypothesized to adsorb on the LNPs surface and interact with lipoprotein receptors for cellular internalization (Morán et al., 2022), as shown in Figure 13 (Zhang et al., 2020). In other cases, antibodies specific to cell surface markers can mediate LNPs internalization (Kampel et al., 2021). With the maturation of the endosomes, the pH decreases below the pKa value, resulting in the increased protonation of the ILs (Younis et al., 2022). The accumulation of accumulation protons and counterions enhances the osmotic transportation of ions and water from the cytoplasm into the endosome. The rapid ionization at pKa values creates proton sponge effects resulting in osmotic swelling (Kalita et al., 2022). The electrostatic interaction between the cationic lipids forming LNPs, and anionic lipids in the plasma membrane can further destabilize the endosomal membrane. During the process, the planar endosomal bilayer structure rearranges to a hexagonal-like shape (Schlich et al., 2021), further bursting of endosomes releasing the siRNA in the cytoplasm following the complete disintegration of LNP structure during the process.

## Current clinical status

Many modified formulations for siRNAs delivery have been demonstrated to have overcome the challenges with membrane penetration and stability. Several candidates for gene silencing are now in the clinical trial pipeline, with the major breakthrough occurring in 2018 with the FDA approval of the first siRNA therapeutics by Alnylam^®^ Pharmaceuticals. The nanoparticle-based siRNA formulation, known as Patisiran (ONPATTRO™) targets transthyretin mRNA and is used for the treatment of polyneuropathy in a hereditary form of transthyretin-mediated (hATTR) amyloidosis (Wood, 2018). As indicated by (de Brito et al., 2022), many drug candidates are still in phase 3 trials. Out of which, Vutrisiran and Inclisiran have been recently approved. Vutrisiran, Givosiran (Givlaari^®^), Lumasiran (Oxluma^®^), and Inclisiran (Leqvio^®^), are the siRNA-based formulations employed to treat hATTR-related polyneuropathy, acute hepatic porphyria, primary hyperoxaluria, and atherosclerotic cardiovascular disorders, respectively (Balwani et al., 2020; Raal et al., 2020; Garrelfs et al., 2021; Aimo et al., 2022). Table 2 summarizes the siRNA formulations under various clinical trials for diverse disorders. No clinical research has now been focused on the siRNA-mediated gene silencing in SARS-CoV-2, but a similar approach can be followed to target divergent sections of the genome of COVID-19 virus leading to the degradation of the viral mRNA sequence. Considering the target specificity and lower side effects, siRNA therapeutics have emerged as a promising therapeutic class (Forgham et al., 2022). However, the fast mutation rate of the viral genome has to be kept in mind with potentially targeting the conserved viral domains with the help of available libraries and computational resources.

**TABLE 2:** Approved and under trial siRNA therapeutics for different diseases.

Name	Manufacturer	Disease	mRNA target	Delivery system	DrugBank accession number	Ref
FDA-approved siRNA therapeutics						
Patisiran (Onpattro^®^)	Alnylam	Polyneuropathy in hereditary transthyretin-mediated amyloidosis	Transthyretin (TTR)	Lipid nanoparticle (DLin-MC3-DMA)	DB14582	Wood, (2018)
Lumasiran (Oxluma^®^)	Alnylam	Primary hyperoxaluria type 1	Hydroxyacid oxidase 1 (HAO1)	GalNAc	DB15935	Garrelfs et al. (2021)
Givosiran (Givlaari^®^)	Alnylam	Acute hepatic porphyria	Aminolevulinic acid synthase 1 (ALAS-1)	GalNAc	DB15066	Balwani et al. (2020)
Inclisiran (Leqvio^®^)	Alnylam-Novartis	Atherosclerotic cardiovascular disease	Proprotein convertase subtilisin/kexin type 9 (PCSK9)	GalNAc	DB14901	Raal et al. (2020)
Vutrisiran (Amvuttra^®^)	Alnylam	Hereditary transthyretin mediated amyloidosis	Transthyretin (TTR)	GalNAc	DB16699	Adams et al. (2022); Aimo et al. (2022)
siRNA therapeutics under clinical trial						
Teprasiran (QPI-1002)	Quark-Norvartis	Prevention of Major Adverse Kidney Events (MAKE)	p53	Naked siRNA molecule	DB15064	Thielmann et al. (2021)
Fitusiran (ALN-AT3)	Alnylam-Sanofi Genzyme	Haemophilia A & B	Antithrombin (AT)	GalNAc	DB15002	Srivastava et al. (2021)
Nedosiran (PHYOX1)	Dicerna-Alnylam	Acute kidney injury	Hepatic lactate dehydrogenase (LDH)	GalNAc	-	Liu et al. (2022)
Cosdosiran (QPI-1007)	Quark	Non-arteritic anterior ischemic optic neuropathy (NAION)	Caspase-2	Naked siRNA molecule	-	Jiang et al. (2021)
Tivanisiran (SYL1001)	Sylentis	Dry eye disease (DED)	Transient Receptor Potential Vanilloid 1 (TRPV1)	Naked siRNA molecule	-	Moreno-Montañés et al. (2018)
Fazirsiran (ARO-AAT/TAK 999)	Arrowhead Pharmaceuticals and Takeda	Liver disease associated with α-1 antitrypsin deficiency (AATD)	Mutant α-1 antitrypsin (Z-AAT)	GalNAc	-	Strnad et al. (2022) (NCT03945292)
ARO-APOC3	Arrowhead Pharmaceuticals	Familial chylomicronemia syndrome (FCS), and Severe Hypertriglyceridemia	Apolipoprotein C-III (APOC3)	GalNAc	-	Watts et al. (2020); Akoumianakis et al. (2021) (NCT05089084)(NCT04720534)
ARO-ANG3	Arrowhead Pharmaceuticals	Treatment of Homozygous Familial Hypercholesterolemia (HOFH), and Mixed Dyslipidemia	Angiopoietin-like 3 (ANGPTL3)	GalNAc	-	Watts et al. (2020); O’Donoghue et al. (2022) (NCT05217667)(NCT04832971)
Olpasiran (AMG 890)	Amgen Pharmaceuticals	Atherosclerotic cardiovascular diseases (ASCVD)	Lipoprotein(a) (Lp(a))	GalNAc	-	O’Donoghue et al. (2022) (NCT05581303)
Revusiran (ALN-TTRSC)	Alnylam	Polyneuropathy in hereditary transthyretin-mediated amyloidosis	Transthyretin (TTR)	GalNAc	DB16309	Judge et al. (2020) (NCT02319005)
SLN360	Silence Therapeutics	Atherosclerotic cardiovascular diseases (ASCVD)	Lipoprotein(a) (Lp(a))	GalNAc	-	Rider et al. (2022) (NCT05537571)
SLN124	Silence Therapeutics	Erythropoiesis and hepatic iron-overload	Transmembrane serine protease 6 (TMPRSS6)	GalNAc	-	Vadolas et al. (2021) (NCT04718844)
ALN-APP	Alnylam-Regeneron	Early onset Alzheimer’s disease and cerebral amyloid angiopathy (CAA)	Amyloid precursor protein (APP)	C16 conjugate technology	-	Akoumianakis et al. (2021) (NCT05231785)
*Cemdisiran* (ALN-CC5)	Alnylam-Regeneron	Complement-mediated diseases, *viz.*, paroxysmal nocturnal hemoglobinuria (PNH), and immunoglobulin A nephropathy (IgAN)	Complement component 5 (C5)	GalNAc	DB16121	Badri et al. (2021) (NCT03841448)
ALN-HBV02/VIR-2218	Alnylam-Vir Biotechnology	Chronic hepatitis B virus (HBV) infection	All viral transcripts and HBV protein	GalNAc and Enhanced stabilization chemistry plus (ESC+) technology	-	Gupta et al. (2021b) (NCT03672188)
ALN-XDH	Alnylam	Gout	Xanthine dehydrogenase (XDH)	GalNAc and Enhanced stabilization chemistry plus (ESC+) technology	-	(NCT05256810)
Zilebesiran (ALN-AGT)	Alnylam	Hypertension	Angiotensinogen	GalNAc and Enhanced stabilization chemistry plus (ESC+) technology	-	(NCT04936035)
ALN-KHK	Alnylam	Type-2 Diabetes Mellitus (T2DM)	ketohexokinase (KHK) or fructokinase	GalNAc and Enhanced stabilization chemistry plus (ESC+) technology	-	NA
Belcesiran (DCR-A1AT)	Dicerna-Alnylam	α-1 antitrypsin (AAT) deficiency-associated liver disease (AATLD)	α-1 antitrypsin (AAT)	GalXC™ RNAi platform based on GalNAc	-	(NCT04764448)
RG6346 (DCR-HBVS)	Dicerna-Roche	Chronic Hepatitis B Virus (HBV) Infection	Conserved S region for the treatment of HBV	GalXC™ RNAi platform based on GalNAc	-	(NCT03772249)(NCT04225715)
DCR-AUD	Dicerna-Alnylam	Alcohol use disorder (AUD)	Aldehyde dehydrogenase 2 family (ALDH2)	GalXC™ RNAi platform based on GalNAc	-	Sasso et al. (2022) (NCT05021640)
AB-729	Arbutus Biopharma	Chronic Hepatitis B Infection	Hepatocytes	GalNAc	-	Phillips et al. (2022) (NCT04980482)
STP705	Sirnaomics	Keloid scarring	Transforming growth factor beta 1 (TGF-β1)/cyclooxygenase-2 (COX-2)	Polypeptide Nanoparticle (PNP)	-	Zhou et al. (2017) (NCT04844840)
ALN-HSD	Alnylam-Regeneron	Non-alcoholic steatohepatitis (NASH)	Hydroxysteroid 17-beta dehydrogenase 13 (HSD17B13)	GalNAc and Enhanced stabilization chemistry plus (ESC+) technology	-	Cui et al. (2021) (NCT04565717)
siG-12D-LODER	Silenseed	Pancreatic Cancer	KRAS (Kirsten rat sarcoma virus)	-	-	Golan et al. (2015) (NCT01676259)
GSK 4532990 (ARO-HSD)	GlaxoSmithKline/Arrowhead Pharmaceuticals	Non-alcoholic steatohepatitis (NASH)	Hydroxysteroid 17-beta dehydrogenase 13 (HSD17B13)	GalNAc	-	(NCT05583344)(NCT04202354)

ClinicalTrials.gov Identifier: NCTⅩⅩⅩⅩⅩⅩⅩⅩ.

## Conclusion

Considering the ever-changing dynamic mutations in the SARS-CoV-2 genome, the demand for well-targeted specific and highly effective therapeutics are needed. Genomic regions of the virus conserved across the variants and sub-variants could be first targeted to inhibit viral entry and replication in the host. Many *in silico* algorithms and web servers have been deployed to design siRNAs following the three golden rules of design, that is Ui-Tei, Amarzguioui and Reynolds. The accessory parameters such as heat capacity (C_P_), melting temperature (T_m_), GC content, and off-target matches are predicted computationally. In a limited set of studies, some validation efforts have been attempted to experimentally verify the silencing efficiency of the computationally designed siRNAs, but this will require more extensive studies to assure confidence in the theoretical designs. Nevertheless, a handful of effective designs are now available that could be tested in a clinical setting, should there be sufficient impetus from clinicians and industrial parties. Further, the designed siRNAs can be formulated with different nanocarriers for practical utility. Lipids-based and polymeric nanoparticles offer the flexibility of conjugation with siRNAs and surface functionalization for targeted delivery. siRNAs-based therapies have been approved for other diseases, but in the case of COVID-19 clinical trials are yet to be undertaken. Along with the efficacy of nano-formulations, other inflammatory responses should be investigated through *in vivo* studies. The COVID-19 treatment, in addition to targeting the viral cause, can be directed to aberrant, excessive inflammation that is ultimately cause of patient exhaustion in clinic. The dosage of siRNAs should be confined to a minimum concentration, preferably below 100 nM at local sites and <1 mg/kg overall for practical translation of delivery systems. The siRNA-based nano-formulations appear to be promising for the therapy of contagious COVID-19 and post-COVID inflammations.

## Abbreviations

siRNA, Small interfering RNA; RNAi, RNA interference; dsRNA, Double-stranded RNAs; RISC, RNA-induced silencing complex; Ago2, Argonaute-2; TRBP, Transactivation response RNA-binding protein; SARS-COV 2, Severe acute respiratory syndrome coronavirus 2; LNP, Lipid nanoparticle; ORF, Open reading frame; hpTCs, Human primary airway-tracheal cells; RdRp, RNA-dependent RNA polymerase; UTR, Untranslated region; LNA, Locked nucleic acid; PLP, Papain-like protease; TNF-α, Tumor necrosis factor-α; IL-6s, Interleukin-6; BLAST, Basic Local Alignment Search Tool; PEG, Poly ethylene glycol; DLin-MC3-DMA, (6Z,9Z,28Z,31Z)-heptatriacont-6,9,28,31-tetraene-19-yl 4-(dimethylamino)butanoate; ALC-0315, [(4-hydroxybutyl)azanediyl]di(hexane-6,1-diyl) bis(2-hexyldecanoate); SM-102, (1-Octylnonyl 8-[(2-hydroxyethyl)[6-oxo-6(undecyloxy)hexyl]amino]-octanoate); DOPE, Dioleoylphosphatidylethanolamine; DSPC, Distearoylphosphatidylcholine; PLA, Poly(lactic acid); PGA, Poly(glycolic acid); PLGA, Poly(lactic-co-glycolide); FDA, Food and Drug Administration; PEI, Polyethyleneimine; ApoE, Apolipoprotein E; DODAP, 1,2-dioleoyl-3-dimethylammonium-propane; RBD, Receptor binding domain; SELEX, Systemic Evolution of Ligands by EXponential enrichment; DMKE, O,O′-dimyristyl-N-lysyl glutamate; SORT, Selective organ targeting; DOTAP, Dioleoyl-3-trimethylammonium propane; hATTR, Hereditary transthyretin amyloidosis.

## References

[B1] Adams D. Tournev I. L. Taylor M. S. Coelho T. Plante-Bordeneuve V. Berk J. L. (2022). Efficacy and safety of vutrisiran for patients with hereditary transthyretin-mediated amyloidosis with polyneuropathy: A randomized clinical trial. Amyloid, 1–9. 10.1080/13506129.2022.2091985 35875890

[B2] Aimo A. Castiglione V. Rapezzi C. Franzini M. Panichella G. Vergaro G. (2022). RNA-targeting and gene editing therapies for transthyretin amyloidosis. Nat. Rev. Cardiol. 19 (10), 655–667. 10.1038/s41569-022-00683-z 35322226

[B3] Akoumianakis I. Zvintzou E. Kypreos K. Filippatos T. D. (2021). ANGPTL3 and Apolipoprotein C-iii as novel lipid-lowering targets. Curr. Atheroscler. Rep. 23 (5), 20–11. 10.1007/s11883-021-00914-7 33694000

[B4] Al-Amin M. D. Bellato F. Mastrotto F. Garofalo M. Malfanti A. Salmaso S. (2020). Dexamethasone loaded liposomes by thin-film hydration and microfluidic procedures: Formulation challenges. Int. J. Mol. Sci. 21 (5), 1611. 10.3390/ijms21051611 32111100 PMC7084920

[B5] Albertsen C. H. Kulkarni J. A. Witzigmann D. Lind M. Petersson K. Simonsen J. B. (2022). The role of lipid components in lipid nanoparticles for vaccines and gene therapy. Adv. Drug Deliv. Rev., 114416. 10.1016/j.addr.2022.114416 35787388 PMC9250827

[B6] Aldosari B. N. Alfagih I. M. Almurshedi A. S. (2021). Lipid nanoparticles as delivery systems for RNA-based vaccines. Pharmaceutics 13 (2), 206. 10.3390/pharmaceutics13020206 33540942 PMC7913163

[B7] Algarni A. Pilkington E. H. Suys E. J. A. Al-Wassiti H. Pouton C. W. Truong N. P. (2022). *In vivo* delivery of plasmid DNA by lipid nanoparticles: The influence of ionizable cationic lipids on organ-selective gene expression. Biomater. Sci. 10 (11), 2940–2952. 10.1039/d2bm00168c 35475455

[B8] Aliabadi H. M. Bahadur K.C. R. Bousoik E. Hall R. Barbarino A. Thapa B. (2020). A systematic comparison of lipopolymers for siRNA delivery to multiple breast cancer cell lines: *In vitro* studies. Acta Biomater. 102, 351–366. 10.1016/j.actbio.2019.11.036 31760224

[B9] Amarzguioui M. Prydz H. (2004). An algorithm for selection of functional siRNA sequences. Biochem. Biophysical Res. Commun. 316 (4), 1050–1058. 10.1016/j.bbrc.2004.02.157 15044091

[B10] Ambike S. Cheng C. C. Feuerherd M. Velkov S. Baldassi D. Afridi S. Q. (2022). Targeting genomic SARS-CoV-2 RNA with siRNAs allows efficient inhibition of viral replication and spread. Nucleic Acids Res. 50 (1), 333–349. 10.1093/nar/gkab1248 34928377 PMC8754636

[B11] Ayyagari V. S. (2022). Design of siRNA molecules for silencing of membrane glycoprotein, nucleocapsid phosphoprotein, and surface glycoprotein genes of SARS-CoV2. J. Genet. Eng. Biotechnol. 20 (1), 65. 10.1186/s43141-022-00346-z 35482116 PMC9047631

[B12] Baden L. R. El Sahly H. M. Essink B. Kotloff K. Frey S. Novak R. (2021). Efficacy and safety of the mRNA-1273 SARS-CoV-2 vaccine. N. Engl. J. Med. 384 (5), 403–416. 10.1056/nejmoa2035389 33378609 PMC7787219

[B13] Badri P. Jiang X. Borodovsky A. Najafian N. Kim J. Clausen V. A. (2021). Pharmacokinetic and pharmacodynamic properties of cemdisiran, an RNAi therapeutic targeting complement component 5, in healthy subjects and patients with paroxysmal nocturnal hemoglobinuria. Clin. Pharmacokinet. 60 (3), 365–378. 10.1007/s40262-020-00940-9 33047216 PMC9203406

[B14] Balwani M. Sardh E. Ventura P. Peiro P. A. Rees D. C. Stolzel U. (2020). Phase 3 trial of RNAi therapeutic givosiran for acute intermittent porphyria. N. Engl. J. Med. 382 (24), 2289–2301. 10.1056/nejmoa1913147 32521132

[B15] Bogaert B. Sauvage F. Guagliardo R. Muntean C. Nguyen V. P. Pottie E. (2022). A lipid nanoparticle platform for mRNA delivery through repurposing of cationic amphiphilic drugs. J. Control. Release 350, 256–270. 10.1016/j.jconrel.2022.08.009 35963467 PMC9401634

[B16] Bunea A. I. Harloff-Helleberg S. Taboryski R. Nielsen H. M. (2020). Membrane interactions in drug delivery: Model cell membranes and orthogonal techniques. Adv. Colloid Interface Sci. 281, 102177. 10.1016/j.cis.2020.102177 32417568

[B17] Cabral H. Makino J. Matsumoto Y. Mi P. Wu H. Nomoto T. (2015). Systemic targeting of lymph node metastasis through the blood vascular system by using size-controlled nanocarriers. ACS Nano 9, 4957–4967. 10.1021/nn5070259 25880444

[B18] Carrasco M. J. Alishetty S. Alameh M. G. Said H. Wright L. Paige M. (2021). Ionization and structural properties of mRNA lipid nanoparticles influence expression in intramuscular and intravascular administration. Commun. Biol. 4 (1), 956. 10.1038/s42003-021-02441-2 34381159 PMC8358000

[B19] Chadar R. Afsana Kesharwani P. (2021). Nanotechnology-based siRNA delivery strategies for treatment of triple negative breast cancer. Int. J. Pharm. 605, 120835. 10.1016/j.ijpharm.2021.120835 34197908

[B20] Chang Y. C. Yang C. Chen Y. Yang C. Chou Y. Chou H. (2022). A siRNA targets and inhibits a broad range of SARS‐CoV‐2 infections including Delta variant. EMBO Mol. Med. 14 (4), e15298. 10.15252/emmm.202115298 35138028 PMC8988202

[B21] Chen W. Feng P. Liu K. Wu M. Lin H. (2020). Computational identification of small interfering RNA targets in SARS-CoV-2. Virol. Sin. 35 (3), 359–361. 10.1007/s12250-020-00221-6 32297156 PMC7157830

[B22] Cheng Q. Wei T. Farbiak L. Johnson L. T. Dilliard S. A. Siegwart D. J. (2020). Selective organ targeting (SORT) nanoparticles for tissue-specific mRNA delivery and CRISPR-Cas gene editing. Nat. Nanotechnol. 15 (4), 313–320. 10.1038/s41565-020-0669-6 32251383 PMC7735425

[B23] Cheng X. Lee R. J. (2016). The role of helper lipids in lipid nanoparticles (LNPs) designed for oligonucleotide delivery. Adv. Drug Deliv. Rev. 99 (), 129–137. 10.1016/j.addr.2016.01.022 26900977

[B24] Chernikov I. V. Vlassov V. V. Chernolovskaya E. L. (2019). Current development of siRNA bioconjugates: From research to the clinic. Front. Pharmacol. 10, 444. 10.3389/fphar.2019.00444 31105570 PMC6498891

[B25] Chowdhury U. F. Sharif Shohan M. U. Hoque K. I. Beg M. A. Sharif Siam M. K. Moni M. A. (2021). A computational approach to design potential siRNA molecules as a prospective tool for silencing nucleocapsid phosphoprotein and surface glycoprotein gene of SARS-CoV-2. Genomics 113 (1), 331–343. 10.1016/j.ygeno.2020.12.021 33321203 PMC7832576

[B26] Corbett K. S. Edwards D. K. Leist S. R. Abiona O. M. Boyoglu-Barnum S. Gillespie R. A. (2020). SARS-CoV-2 mRNA vaccine design enabled by prototype pathogen preparedness. Nature 586 (7830), 567–571. 10.1038/s41586-020-2622-0 32756549 PMC7581537

[B27] Cui H. Zhu X. Li S. Wang P. Fang J. (2021). Liver-targeted delivery of oligonucleotides with N-acetylgalactosamine conjugation. ACS Omega 6 (25), 16259–16265. 10.1021/acsomega.1c01755 34235295 PMC8246477

[B28] Dar S. A. Gupta A. K. Thakur A. Kumar M. (2016). SMEpred workbench: A web server for predicting efficacy of chemicallymodified siRNAs. RNA Biol. 13 (11), 1144–1151. 10.1080/15476286.2016.1229733 27603513 PMC5100349

[B29] Dar S. A. Thakur A. Qureshi A. Kumar M. (2016). siRNAmod: A database of experimentally validated chemically modified siRNAs. Sci. Rep. 6 (1), 20031. 10.1038/srep20031 26818131 PMC4730238

[B30] de Brito E. C. D. Frederico A. Azamor T. Melgaco J. da Costa Neves P. Bom A. (2022). Biotechnological evolution of siRNA molecules: From bench tool to the refined drug. Pharm. (Basel) 15 (5), 575. 10.3390/ph15050575 PMC9146980 35631401

[B31] DiChiacchio L. Sloma M. F. Mathews D. H. (2016). AccessFold: Predicting RNA–RNA interactions with consideration for competing self-structure. Bioinformatics 32 (7), 1033–1039. 10.1093/bioinformatics/btv682 26589271 PMC4907385

[B32] Dilliard S. A. Cheng Q. Siegwart D. J. (2021). On the mechanism of tissue-specific mRNA delivery by selective organ targeting nanoparticles. Proc. Natl. Acad. Sci. U. S. A. 118 (52), e2109256118. 10.1073/pnas.2109256118 34933999 PMC8719871

[B33] Dobrowolski C. Paunovska K. Hatit M. Z. C. Lokugamage M. P. Dahlman J. E. (2021). Therapeutic RNA delivery for COVID and other diseases. Adv. Healthc. Mater 10 (15), e2002022. 10.1002/adhm.202002022 33661555 PMC7995096

[B34] Dormenval C. Lokras A. Cano-Garcia G. Wadhwa A. Thanki K. Rose F. (2019). Identification of factors of importance for spray drying of small interfering RNA-loaded lipidoid-polymer hybrid nanoparticles for inhalation. Pharm. Res. 36 (10), 142. 10.1007/s11095-019-2663-y 31376020

[B35] Dymek M. Sikora E. (2022). Liposomes as biocompatible and smart delivery systems-the current state. Adv. Colloid Interface Sci. 309, 102757. 10.1016/j.cis.2022.102757 36152374

[B36] Evers M. J. van de Wakker S. I. de Groot E. M. de Jong O. G. Gitz‐Francois J. J. J. Seinen C. S. (2022). Functional siRNA delivery by extracellular vesicle–liposome hybrid nanoparticles. J. Adv. Healthc. Mater. 11 (5), 2101202. 10.1002/adhm.202101202 PMC11468224 34382360

[B37] Ferraresso F. Strilchuk A. W. Juang L. J. Poole L. G. Luyendyk J. P. Kastrup C. J. (2022). Comparison of DLin-MC3-DMA and ALC-0315 for siRNA delivery to hepatocytes and hepatic stellate cells. Mol. Pharm. 19 (7), 2175–2182. 10.1021/acs.molpharmaceut.2c00033 35642083 PMC9621687

[B38] Forgham H. Kakinen A. Qiao R. Davis T. P. (2022). Keeping up with the COVID's—could siRNA‐based antivirals be a part of the answer? Exploration. Exploration, 20220012. 10.1002/EXP.20220012 35941991 PMC9349879

[B39] Friedrich M. Pfeifer G. Binder S. Aigner A. Vollmer Barbosa P. Makert G. R. (2022). Selection and validation of siRNAs preventing uptake and replication of SARS-CoV-2. Front. Bioeng. Biotechnol. 10, 801870. 10.3389/fbioe.2022.801870 35309990 PMC8925020

[B40] Gallicano G. I. Casey J. L. Fu J. Mahapatra S. (2022). Molecular targeting of vulnerable RNA sequences in SARS CoV-2: Identifying clinical feasibility. Gene Ther. 29 (5), 304–311. 10.1038/s41434-020-00210-0 33184504 PMC7659899

[B41] Garrelfs S. F. Frishberg Y. Hulton S. A. Koren M. J. O’Riordan W. D. Cochat P. (2021). Lumasiran, an RNAi therapeutic for primary hyperoxaluria type 1. J N. Engl. J. Med. 384 (13), 1216–1226. 10.1056/nejmoa2021712 33789010

[B42] Gilham D. Lehner R. (2005). Techniques to measure lipase and esterase activity *in vitro* . Methods 36, 139–147. 10.1016/j.ymeth.2004.11.003 15893936

[B43] Golan T. Khvalevsky E. Z. Hubert A. Gabai R. M. Hen N. Segal A. (2015). RNAi therapy targeting KRAS in combination with chemotherapy for locally advanced pancreatic cancer patients. Oncotarget 6 (27), 24560–24570. 10.18632/oncotarget.4183 26009994 PMC4695206

[B44] Gruber A. R. Bernhart S. H. Lorenz R. (2015). “The ViennaRNA web services,” in RNA bioinformatics. Editor Picardi E. (New York, NY: Springer New York), 307–326. 10.1007/978-1-4939-2291-8_19 25577387

[B45] Gupta A. Anand A. Jain N. Goswami S. Anantharaj A. Patil S. (2021). A novel G-quadruplex aptamer-based spike trimeric antigen test for the detection of SARS-CoV-2. Mol. Ther. - Nucleic Acids 26, 321–332. 10.1016/j.omtn.2021.06.014 34188971 PMC8223116

[B46] Gupta N. Bharti Rai D. Jangid A. K. Pooja D. Kulhari H. (2019). Nanomaterials-based siRNA delivery: Routes of administration, hurdles and role of nanocarriers. Nanotechnol. Mod. Animal Biotechnol., 67–114. 10.1007/978-981-13-6004-6_3

[B47] Gupta S. V. Fanget M. C. MacLauchlin C. Clausen V. A. Li J. Cloutier D. (2021). Clinical and preclinical single-dose pharmacokinetics of VIR-2218, an RNAi therapeutic targeting HBV infection. Drugs R. D. 21 (4), 455–465. 10.1007/s40268-021-00369-w 34741731 PMC8602582

[B48] Han Q. Chen G. Wang J. Jee D. Li W. X. Lai E. C. (2020). Mechanism and function of antiviral RNA interference in mice. mBio 11 (4), 032788–e3319. 10.1128/mbio.03278-19 PMC7407090 32753500

[B49] Has C. Sunthar P. (2020). A comprehensive review on recent preparation techniques of liposomes. J. Liposome Res. 30 (4), 336–365. 10.1080/08982104.2019.1668010 31558079

[B50] Hasan M. Ashik A. I. Chowdhury M. B. Tasnim A. T. Nishat Z. S. Hossain T. (2021). Computational prediction of potential siRNA and human miRNA sequences to silence orf1ab associated genes for future therapeutics against SARS-CoV-2. Inf. Med. Unlocked 24, 100569. 10.1016/j.imu.2021.100569 PMC8028608 33846694

[B51] Hassett K. J. Benenato K. E. Jacquinet E. Lee A. Woods A. Yuzhakov O. (2019). Optimization of lipid nanoparticles for intramuscular administration of mRNA vaccines. Mol. Ther. Nucleic Acids 15, 1–11. 10.1016/j.omtn.2019.01.013 30785039 PMC6383180

[B52] Hou X. Zaks T. Langer R. Dong Y. (2021). Lipid nanoparticles for mRNA delivery. Nat. Rev. Mater 6 (12), 1078–1094. 10.1038/s41578-021-00358-0 34394960 PMC8353930

[B53] Hu B. Guo H. Zhou P. Shi Z. L. (2021). Characteristics of SARS-CoV-2 and COVID-19. Nat. Rev. Microbiol. 19 (3), 141–154. 10.1038/s41579-020-00459-7 33024307 PMC7537588

[B54] Hu B. Zhong L. Weng Y. Peng L. Huang Y. Zhao Y. (2020). Therapeutic siRNA: State of the art. Signal Transduct. Target. Ther. 5 (1), 101. 10.1038/s41392-020-0207-x 32561705 PMC7305320

[B55] Hung W. C. Lee M. T. Chen F. Y. Huang H. W. (2007). The condensing effect of cholesterol in lipid bilayers. Biophys. J. 92, 3960–3967. 10.1529/biophysj.106.099234 17369407 PMC1868968

[B56] Ichihara M. Murakumo Y. Masuda A. Matsuura T. Asai N. Jijiwa M. (2007). Thermodynamic instability of siRNA duplex is a prerequisite for dependable prediction of siRNA activities. Nucleic Acids Res. 35 (18), e123. 10.1093/nar/gkm699 17884914 PMC2094068

[B57] Idris A. Davis A. Supramaniam A. Acharya D. Kelly G. Tayyar Y. (2021). A SARS-CoV-2 targeted siRNA-nanoparticle therapy for COVID-19. Mol. Ther. 29 (7), 2219–2226. 10.1016/j.ymthe.2021.05.004 33992805 PMC8118699

[B58] Jayaraman M. Ansell S. M. Mui B. L. Tam Y. K. Chen J. Du X. (2012). Maximizing the potency of siRNA lipid nanoparticles for hepatic gene silencing *in vivo* . Angew. Chem. Int. Ed. Engl. 51 (34), 8657–8661. 10.1002/ange.201203263 PMC3470698 22782619

[B59] Jiang J. Zhang X. Tang Y. Li S. Chen J. (2021). Progress on ocular siRNA gene-silencing therapy and drug delivery systems. Fundam. Clin. Pharmacol. 35 (1), 4–24. 10.1111/fcp.12561 32298491

[B60] Jo M. Park K. M. Park J. Y. Yu H. Choi S. J. Chang P. S. (2020). Microfluidic assembly of mono-dispersed liposome and its surface modification for enhancing the colloidal stability. Colloids Surfaces a-Physicochemical Eng. Aspects 586, 124202. 10.1016/j.colsurfa.2019.124202

[B61] Judge D. P. Kristen A. V. Grogan M. Maurer M. S. Falk R. H. Hanna M. (2020). Phase 3 multicenter study of revusiran in patients with hereditary transthyretin-mediated (hATTR) amyloidosis with cardiomyopathy (ENDEAVOUR). J. Cardiovasc. drugs Ther. 34 (3), 357–370. 10.1007/s10557-019-06919-4 PMC7242280 32062791

[B62] Kalita T. Dezfouli S. A. Pandey L. M. Uludag H. (2022). siRNA functionalized lipid nanoparticles (LNPs) in management of diseases. J. Pharm. 14 (11), 2520. 10.3390/pharmaceutics14112520 PMC9694336 36432711

[B63] Kampel L. Goldsmith M. Ramishetti S. Veiga N. Rosenblum D. Gutkin A. (2021). Therapeutic inhibitory RNA in head and neck cancer via functional targeted lipid nanoparticles. J. Control Release 337, 378–389. 10.1016/j.jconrel.2021.07.034 34303750

[B64] Karimov M. Schulz M. Kahl T. Noske S. Kubczak M. Gockel I. (2021). Tyrosine-modified linear PEIs for highly efficacious and biocompatible siRNA delivery *in vitro* and *in vivo* . Nanomedicine 36, 102403. 10.1016/j.nano.2021.102403 33932594

[B65] Khaitov M. Nikonova A. Shilovskiy I. Kozhikhova K. Kofiadi I. Vishnyakova L. (2021). Silencing of SARS‐CoV‐2 with modified siRNA‐peptide dendrimer formulation. Allergy 76 (9), 2840–2854. 10.1111/all.14850 33837568 PMC8251148

[B66] Khanali J. Azangou-Khyavy M. Asaadi Y. Jamalkhah M. Kiani J. (2021). Nucleic acid-based treatments against COVID-19: Potential efficacy of aptamers and siRNAs, Front Microbiol 12, 758948. 10.3389/fmicb.2021.758948 34858370 PMC8630580

[B67] Khare P. Dave K. M. Kamte Y. S. Manoharan M. A. O’Donnell L. A. Manickam D. S. (2022). Development of lipidoid nanoparticles for siRNA delivery to neural cells. J AAPS J. 24 (1), 8–17. 10.1208/s12248-021-00653-2 PMC8648339 34873640

[B68] Kibbe W. A. (2007). OligoCalc: An online oligonucleotide properties calculator. Nucleic Acids Res. 35, W43–W46. (Web Server). 10.1093/nar/gkm234 17452344 PMC1933198

[B69] Koitabashi K. Nagumo H. Nakao M. Machida T. Yoshida K. Sakai-Kato K. (2021). Acidic pH induced changes in lipid nanoparticle membrane packing. Biochim. Biophys. Acta - Biomembr. 1863, 183627. 10.1016/j.bbamem.2021.183627 33901441

[B70] Koltover I. Salditt T. Rädler J. O. Safinya C. R. (1998). An inverted hexagonal phase of cationic liposome-DNA complexes related to DNA release and delivery. Science 281 (5373), 78–81. 10.1126/science.281.5373.78 9651248

[B71] Kon E. Elia U. Peer D. (2022). Principles for designing an optimal mRNA lipid nanoparticle vaccine. Curr. Opin. Biotechnol. 73, 329–336. 10.1016/j.copbio.2021.09.016 34715546 PMC8547895

[B72] Kulkarni J. A. Cullis P. R. van der Meel R. (2018). Lipid nanoparticles enabling gene therapies: From concepts to clinical utility. Nucleic Acid. Ther. 28 (3), 146–157. 10.1089/nat.2018.0721 29683383

[B73] Lechanteur A. Sanna V. Duchemin A. Evrard B. Mottet D. Piel G. (2018). Cationic liposomes carrying siRNA: Impact of lipid composition on physicochemical properties, cytotoxicity and endosomal escape. Nanomater. (Basel) 8 (5), 270. 10.3390/nano8050270 PMC5977284 29695068

[B74] Lee J. Kim D. Byun J. Wu Y. Park J. Oh Y. K. (2022). *In vivo* fate and intracellular trafficking of vaccine delivery systems. Adv. Drug Deliv. Rev. 186, 114325. 10.1016/j.addr.2022.114325 35550392 PMC9085465

[B75] Li J. Zhang Z. Gu J. Stacey H. D. Ang J. C. Capretta A. (2021). Diverse high-affinity DNA aptamers for wild-type and B.1.1.7 SARS-CoV-2 spike proteins from a pre-structured DNA library. Nucleic Acids Res. 49 (13), 7267–7279. 10.1093/nar/gkab574 34232998 PMC8287928

[B76] Liu A. Zhao J. Shah M. Migliorati J. M. Tawfik S. M. Bahal R. (2022). Nedosiran, a candidate siRNA drug for the treatment of primary hyperoxaluria: Design, development, and clinical studies. ACS Pharmacol. Transl. Sci. 5 (11), 1007–1016. 10.1021/acsptsci.2c00110 36407951 PMC9667536

[B77] Lu Z. J. Gloor J. W. Mathews D. H. (2009). Improved RNA secondary structure prediction by maximizing expected pair accuracy. RNA 15 (10), 1805–1813. 10.1261/rna.1643609 19703939 PMC2743040

[B78] Luten J. van Nostrum C. F. De Smedt S. C. Hennink W. E. (2008). Biodegradable polymers as non-viral carriers for plasmid DNA delivery. J. Control. Release 126, 97–110. 10.1016/j.jconrel.2007.10.028 18201788

[B79] Ly H. H. Daniel S. Soriano S. K. V. Kis Z. Blakney A. K. (2022). Optimization of lipid nanoparticles for saRNA expression and cellular activation using a design-of-experiment approach. Mol. Pharm. 19 (6), 1892–1905. 10.1021/acs.molpharmaceut.2c00032 35604765 PMC9176215

[B80] Maier M. A. Jayaraman M. Matsuda S. Liu J. Barros S. Querbes W. (2013). Biodegradable lipids enabling rapidly eliminated lipid nanoparticles for systemic delivery of RNAi therapeutics. Mol. Ther. 21 (8), 1570–1578. 10.1038/mt.2013.124 23799535 PMC3734658

[B81] Markham N. R. Zuker M. (2005). DINAMelt web server for nucleic acid melting prediction. Nucleic Acids Res. 33, W577–W581. (Web Server). 10.1093/nar/gki591 15980540 PMC1160267

[B82] Masatoshi M. Okada Y. Uno S. Niwa A. Ishida A. Tani H. (2022). Production of sirna-loaded lipid nanoparticles using a microfluidic device. J. Vis. Exp., 62999. 10.3791/62999 35404350

[B83] Mathews D. H. (2010). “Using OligoWalk to identify efficient siRNA sequences,” in RNA therapeutics. Editor Sioud M. (Totowa, NJ: Humana Press), 107–119. 10.1007/978-1-60761-657-3_8 20387146

[B84] Medeiros I. G. Khayat A. S. Stransky B. Santos S. Assumpcao P. de Souza J. E. S. (2021). A small interfering RNA (siRNA) database for SARS-CoV-2. Sci. Rep. 11 (1), 8849. 10.1038/s41598-021-88310-8 33893357 PMC8065152

[B85] Montazeri Aliabadi H. Totonchy J. Mahdipoor P. Parang K. Uludag H. (2021). Suppression of human coronavirus 229E infection in lung fibroblast cells via RNA interference. J Front. Nanotechnol. 3, 34. 10.3389/fnano.2021.670543

[B86] Morán L. Maximilian Woitok M. Bartneck M. Javier Cubero F. (2022). Hepatocyte-directed delivery of lipid-encapsulated small interfering RNA. Methods Mol. Biol. 2544, 95–106. 10.1007/978-1-0716-2557-6_6 36125712

[B87] Moreno-Montañés J. Bleau A.-M. Jimenez A. I. (2018). Tivanisiran, a novel siRNA for the treatment of dry eye disease. Expert Opin. Investigational Drugs 27 (4), 421–426. 10.1080/13543784.2018.1457647 29569947

[B88] Muhseen Z. T. Hameed A. R. Al-Hasani H. M. Tahir ul Qamar M. Li G. (2020). Promising terpenes as SARS-CoV-2 spike receptor-binding domain (RBD) attachment inhibitors to the human ACE2 receptor: Integrated computational approach. J. Mol. Liq. 320, 114493. 10.1016/j.molliq.2020.114493 33041407 PMC7538380

[B89] Naito Y. Yoshimura J. Morishita S. Ui-Tei K. (2009). siDirect 2.0: updated software for designing functional siRNA with reduced seed-dependent off-target effect. BMC Bioinforma. 10 (1), 392. 10.1186/1471-2105-10-392 PMC2791777 19948054

[B90] Nezhad M. S. J. B. (2022). Poly (beta‐amino ester) as an *in vivo* nanocarrier for therapeutic nucleic acids. J. Biotechnol. Bioeng. 120, 95–113. 10.1002/bit.28269 36266918

[B91] Niktab I. Haghparast M. Beigi M. H. Megraw T. L. Kiani A. Ghaedi K. (2021). Design of advanced siRNA therapeutics for the treatment of COVID-19. Meta Gene 29, 100910. 10.1016/j.mgene.2021.100910 33996501 PMC8106235

[B92] Nogueira E. Freitas J. Loureiro A. Nogueira P. Gomes A. C. Preto A. (2017). Neutral PEGylated liposomal formulation for efficient folate-mediated delivery of MCL1 siRNA to activated macrophages. Colloids Surf. B Biointerfaces 155, 459–465. 10.1016/j.colsurfb.2017.04.023 28472749

[B93] O’Donoghue M. L. Rosenson R. S. Gencer B. Lopez J. A. G. Lepor N. E. Baum S. J. (2022). Small interfering RNA to reduce lipoprotein (a) in cardiovascular disease. J N. Engl. J. Med. 387 (20), 1855–1864. 10.1056/nejmoa2211023 36342163

[B94] Panda K. Alagarasu K. Parashar D. (2021). Prediction of potential small interfering RNA molecules for silencing of the spike gene of SARS-CoV-2. Indian J. Med. Res. 153 (1), 182. 10.4103/ijmr.ijmr_2855_20 33818475 PMC8184069

[B95] Pandey A. K. Verma S. (2021). An *in silico* analysis of effective siRNAs against COVID‐19 by targeting the leader sequence of SARS‐CoV‐2. Adv. CELL GENE Ther. 4 (2), e107. 10.1002/acg2.107 33786418 PMC7995175

[B96] Paramasivam P. Franke C. Stoter M. Hoijer A. Bartesaghi S. Sabirsh A. (2022). Endosomal escape of delivered mRNA from endosomal recycling tubules visualized at the nanoscale. J. Cell Biol. 221 (2), e202110137. 10.1083/jcb.202110137 34882187 PMC8666849

[B97] Pei Y. Bao Y. Sacchetti C. Brady J. Gillard K. Yu H. (2022). Synthesis and bioactivity of readily hydrolysable novel cationic lipids for potential lung delivery application of mRNAs. Chem. Phys. Lipids 243, 105178. 10.1016/j.chemphyslip.2022.105178 35122738 PMC9749011

[B98] Phillips S. Jagatia R. Chokshi S. (2022). Novel therapeutic strategies for chronic Hepatitis B. Virulence 13 (1), 1111–1132. 10.1080/21505594.2022.2093444 35763282 PMC9272843

[B99] Prata C. A. Zhao Y. Barthelemy P. Li Y. Luo D. McIntosh T. J. (2004). Charge-reversal amphiphiles for gene delivery. J. Am. Chem. Soc. 126, 12196–12197. 10.1021/ja0474906 15453715

[B100] Qureshi A. Thakur N. Kumar M. (2013). VIRsiRNApred: A web server for predicting inhibition efficacy of siRNAs targeting human viruses. J. Transl. Med. 11 (1), 305. 10.1186/1479-5876-11-305 24330765 PMC3878835

[B101] Raal F. J. Kallend D. Ray K. K. Turner T. Koenig W. Wright R. S. (2020). Inclisiran for the treatment of heterozygous familial hypercholesterolemia. N. Engl. J. Med. 382 (16), 1520–1530. 10.1056/nejmoa1913805 32197277

[B102] Rajappan K. Tanis S. P. Mukthavaram R. Roberts S. Nguyen M. Tachikawa K. (2020). Property-driven design and development of lipids for efficient delivery of siRNA. J. Med. Chem. 63 (21), 12992–13012. 10.1021/acs.jmedchem.0c01407 33119286

[B103] Ramachandran S. Satapathy S. R. Dutta T. (2022). Delivery strategies for mRNA vaccines. Pharm. Med. 36 (1), 11–20. 10.1007/s40290-021-00417-5 PMC8801198 35094366

[B104] Ren H. He Y. Liang J. Cheng Z. Zhang M. Zhu Y. (2019). Role of liposome size, surface charge, and PEGylation on rheumatoid arthritis targeting therapy. ACS Appl. Mater Interfaces 11 (22), 20304–20315. 10.1021/acsami.8b22693 31056910

[B105] Reynolds A. Leake D. Boese Q. Scaringe S. Marshall W. S. Khvorova A. (2004). Rational siRNA design for RNA interference. Nat. Biotechnol. 22 (3), 326–330. 10.1038/nbt936 14758366

[B106] Rider D. A. Eisermann M. Loffler K. Aleku M. Swerdlow D. I. Dames S. (2022). Pre-clinical assessment of SLN360, a novel siRNA targeting LPA, developed to address elevated lipoprotein (a) in cardiovascular disease. Atherosclerosis 349, 240–247. 10.1016/j.atherosclerosis.2022.03.029 35400495

[B107] Saadati F. Cammarone S. Ciufolini M. A. (2022). A route to lipid ALC-0315: A key component of a COVID-19 mRNA vaccine. Chemistry 28 (48), e202200906. 10.1002/chem.202200906 35665545 PMC9348069

[B108] Sabnis S. Kumarasinghe E. S. Salerno T. Mihai C. Ketova T. Senn J. J. (2018). A novel amino lipid series for mRNA delivery: Improved endosomal escape and sustained pharmacology and safety in non-human primates. Mol. Ther. 26, 1509–1519. 10.1016/j.ymthe.2018.03.010 29653760 PMC5986714

[B109] Saeed R. M. Abdullah M. Ahram M. Taha M. O. (2021). Novel ellipsoid chitosan-phthalate lecithin nanoparticles for siRNA delivery. Front. Bioeng. Biotechnol. 9, 695371. 10.3389/fbioe.2021.695371 34395401 PMC8355739

[B110] Saify Nabiabad H. Amini M. Demirdas S. (2022). Specific delivering of RNAi using spike's aptamer‐functionalized lipid nanoparticles for targeting SARS‐CoV‐2: A strong anti‐covid drug in a clinical case study. Chem. Biol. Drug Des. 99 (2), 233–246. 10.1111/cbdd.13978 34714580 PMC8653378

[B111] Sajid M. I. Moazzam M. Cho Y. Kato S. Xu A. Way J. J. (2021). siRNA therapeutics for the therapy of COVID-19 and other coronaviruses. Mol. Pharm. 18 (6), 2105–2121. 10.1021/acs.molpharmaceut.0c01239 33945284

[B112] Sasso J. M. Ambrose B. J. B. Tenchov R. Datta R. S. Basel M. T. DeLong R. K. (2022). The progress and promise of RNA Medicine─ an arsenal of targeted treatments. J. Med. Chem. 65, 6975–7015. 10.1021/acs.jmedchem.2c00024 35533054 PMC9115888

[B113] Sato Y. Hashiba K. Sasaki K. Maeki M. Tokeshi M. Harashima H. (2019). Understanding structure-activity relationships of pH-sensitive cationic lipids facilitates the rational identification of promising lipid nanoparticles for delivering siRNAs *in vivo* . J. Control. Release 295, 140–152. 10.1016/j.jconrel.2019.01.001 30610950

[B114] Sato Y. Note Y. Maeki M. Kaji N. Baba Y. Tokeshi M. (2016). Elucidation of the physicochemical properties and potency of siRNA-loaded small-sized lipid nanoparticles for siRNA delivery. J. Control. Release 229, 48–57. 10.1016/j.jconrel.2016.03.019 26995758

[B115] Schlich M. Palomba R. Costabile G. Mizrahy S. Pannuzzo M. Peer D. (2021). Cytosolic delivery of nucleic acids: The case of ionizable lipid nanoparticles. Bioeng. Transl. Med. 6 (2), e10213. 10.1002/btm2.10213 33786376 PMC7995196

[B116] Schmitz A. Weber A. Bayin M. Breuers S. Fieberg V. Famulok M. (2021). A SARS‐CoV‐2 spike binding DNA aptamer that inhibits pseudovirus infection by an RBD‐independent mechanism**. Angew. Chem. Int. Ed. 60 (18), 10367–10373. 10.1002/ange.202100316 PMC8251191 33683787

[B117] Schoenmaker L. Witzigmann D. Kulkarni J. A. Verbeke R. Kersten G. Jiskoot W. (2021). mRNA-lipid nanoparticle COVID-19 vaccines: Structure and stability. Int. J. Pharm. 601, 120586. 10.1016/j.ijpharm.2021.120586 33839230 PMC8032477

[B118] Sebastiani F. Yanez Arteta M. Lerche M. Porcar L. Lang C. Bragg R. A. (2021). Apolipoprotein E binding drives structural and compositional rearrangement of mRNA-containing lipid nanoparticles. ACS Nano 15 (4), 6709–6722. 10.1021/acsnano.0c10064 33754708 PMC8155318

[B119] Shah R. M. Eldridge D. S. Palombo E. A. Harding I. H. (2022). Stability mechanisms for microwave-produced solid lipid nanoparticles. Colloids Surf. A Physicochem. Eng. Asp. 643, 128774. 10.1016/j.colsurfa.2022.128774

[B120] Shawan M. M. A. K. Sharma A. R. Bhattacharya M. Mallik B. Akhter F. Shakil M. S. (2021). Designing an effective therapeutic siRNA to silence RdRp gene of SARS-CoV-2. Infect. Genet. Evol. 93, 104951. 10.1016/j.meegid.2021.104951 34089909 PMC8170914

[B121] Sievers F. Higgins D. G. (2018). Clustal omega for making accurate alignments of many protein sequences: Clustal omega for many protein sequences. Protein Sci. 27 (1), 135–145. 10.1002/pro.3290 28884485 PMC5734385

[B122] Srivastava A. Rangarajan S. Kavakli K. Klamroth R. Kenet G. Khoo L. (2021). Fitusiran, an Investigational siRNA therapeutic targeting Antithrombin for the Treatment of hemophilia: First Results from a phase 3 Study to evaluate Efficacy and Safety in People with Hemophilia a or B without inhibitors (ATLAS-A/B) . Blood 138 (2), LBA3. . 10.1182/blood-2021-155018

[B123] Strnad P. Mandorfer M. Choudhury G. Griffiths W. Trautwein C. Loomba R. (2022). Fazirsiran for liver disease associated with alpha1-antitrypsin deficiency. J N. Engl. J. Med. 387 (6), 514–524. 10.1056/nejmoa2205416 35748699

[B124] Suk J. S. Xu Q. Kim N. Hanes J. Ensign L. M. (2016). PEGylation as a strategy for improving nanoparticle-based drug and gene delivery. Adv. Drug Deliv. Rev. 99, 28–51. 10.1016/j.addr.2015.09.012 26456916 PMC4798869

[B125] Suzuki Y. Hyodo K. Suzuki T. Tanaka Y. Kikuchi H. Ishihara H. (2017). Biodegradable lipid nanoparticles induce a prolonged RNA interference-mediated protein knockdown and show rapid hepatic clearance in mice and nonhuman primates. Int. J. Pharm. 519, 34–43. 10.1016/j.ijpharm.2017.01.016 28089936

[B126] Suzuki Y. Ishihara H. (2021). Difference in the lipid nanoparticle technology employed in three approved siRNA (Patisiran) and mRNA (COVID-19 vaccine) drugs. Drug Metab. Pharmacokinet. 41, 100424. 10.1016/j.dmpk.2021.100424 34757287 PMC8502116

[B127] Suzuki Y. Miyazaki T. Muto H. Kubara K. Mukai Y. Watari R. (2022). Design and lyophilization of lipid nanoparticles for mRNA vaccine and its robust immune response in mice and nonhuman primates. Mol. Ther. Nucleic Acids 30, 226–240. 10.1016/j.omtn.2022.09.017 36187052 PMC9508692

[B128] Syama K. Jakubek Z. J. Chen S. Zaifman J. Tam Y. Y. C. Zou S. (2022). Development of lipid nanoparticles and liposomes reference materials (II): Cytotoxic profiles. Sci. Rep. 12 (1), 18071. 10.1038/s41598-022-23013-2 36302886 PMC9610362

[B129] Tafer H. Ameres S. L. Obernosterer G. Gebeshuber C. A. Schroeder R. Martinez J. (2008). The impact of target site accessibility on the design of effective siRNAs. Nat. Biotechnol. 26 (5), 578–583. 10.1038/nbt1404 18438400

[B130] Tenchov R. Bird R. Curtze A. E. Zhou Q. (2021). Lipid nanoparticles─ from liposomes to mRNA vaccine delivery, a landscape of research diversity and advancement. J. ACS nano 15 (11), 16982–17015. 10.1021/acsnano.1c04996 34181394

[B131] Terada T. Kulkarni J. A. Huynh A. Chen S. van der Meel R. Tam Y. Y. C. (2021). Characterization of lipid nanoparticles containing ionizable cationic lipids using design-of-experiments approach. Langmuir 37 (3), 1120–1128. 10.1021/acs.langmuir.0c03039 33439022

[B132] Thi Nhu Thao T. Labroussaa F. Ebert N. V’kovski P. Stalder H. Portmann J. (2020). Rapid reconstruction of SARS-CoV-2 using a synthetic genomics platform. Nature 582 (7813), 561–565. 10.1038/s41586-020-2294-9 32365353

[B133] Thielmann M. Corteville D. Szabo G. Swaminathan M. Lamy A. Lehner L. J. (2021). Teprasiran, a small interfering RNA, for the prevention of acute kidney injury in high-risk patients undergoing cardiac surgery: A randomized clinical study. Circulation 144 (14), 1133–1144. 10.1161/circulationaha.120.053029 34474590 PMC8487715

[B134] Tian G. Pan R. Zhang B. Qu M. Lian B. Jiang H. (2019). Liver-targeted combination therapy basing on glycyrrhizic acid-modified DSPE-PEG-PEI nanoparticles for co-delivery of doxorubicin and Bcl-2 siRNA. Front. Pharmacol. 10, 4. 10.3389/fphar.2019.00004 30723405 PMC6349772

[B135] Tolksdorf B. Nie C. Niemeyer D. Rohrs V. Berg J. Lauster D. (2021). Inhibition of SARS-CoV-2 replication by a small interfering RNA targeting the leader sequence. Viruses 13 (10), 2030. 10.3390/v13102030 34696460 PMC8539227

[B136] Ui-Tei K. (2004). Guidelines for the selection of highly effective siRNA sequences for mammalian and chick RNA interference. Nucleic Acids Res. 32 (3), 936–948. 10.1093/nar/gkh247 14769950 PMC373388

[B137] Ullah A. Qazi J. Rahman L. Kanaras A. G. Khan W. S. Hussain I. (2020). Nanoparticles‐assisted delivery of antiviral‐siRNA as inhalable treatment for human respiratory viruses: A candidate approach against SARS‐COV‐2. Nano Sel. 1 (6), 612–621. 10.1002/nano.202000125 34485978 PMC7675679

[B138] Uludağ H. Parent K. Aliabadi H. M. Haddadi A. (2020). Prospects for RNAi therapy of COVID-19. Front. Bioeng. Biotechnol. 8, 916. 10.3389/fbioe.2020.00916 32850752 PMC7409875

[B139] Urits I. Swanson D. Swett M. C. Patel A. Berardino K. Amgalan A. (2021). Correction to: A review of patisiran (ONPATTRO®) for the treatment of polyneuropathy in people with hereditary transthyretin amyloidosis. Neurol. Ther. 10 (1), 407. 10.1007/s40120-020-00228-x 33433892 PMC8140161

[B140] Vadolas J. Ng G. Z. Kysenius K. Crouch P. J. Dames S. Eisermann M. (2021). SLN124, a GalNac-siRNA targeting transmembrane serine protease 6, in combination with deferiprone therapy reduces ineffective erythropoiesis and hepatic iron-overload in a mouse model of beta-thalassaemia. Br. J. Haematol. 194 (1), 200–210. 10.1111/bjh.17428 33942901 PMC8359948

[B141] V’kovski P. Kratzel A. Steiner S. Stalder H. Thiel V. (2021). Coronavirus biology and replication: Implications for SARS-CoV-2. Nat. Rev. Microbiol. 19 (3), 155–170. 10.1038/s41579-020-00468-6 33116300 PMC7592455

[B142] Walsh E. E. Frenck R. W. Falsey A. R. Kitchin N. Absalon J. Gurtman A. (2020). Safety and immunogenicity of two RNA-based covid-19 vaccine candidates. N. Engl. J. Med. 383 (25), 2439–2450. 10.1056/nejmoa2027906 33053279 PMC7583697

[B143] Wang X. Liu S. Sun Y. Yu X. Lee S. M. Cheng Q. (2022). Preparation of selective organ-targeting (SORT) lipid nanoparticles (LNPs) using multiple technical methods for tissue-specific mRNA delivery. Nat. Protoc. 18, 265–291. 10.1038/s41596-022-00755-x 36316378 PMC9888002

[B144] Watts G. F. Schwabe C. Scott R. Gladding P. Sullivan D. Baker J. (2020). Pharmacodynamic effect of ARO-ANG3, an investigational RNA interference targeting hepatic angiopoietin-like protein 3, in patients with hypercholesterolemia. Circulation 142 (3), A15751.

[B145] Wood H. (2018). FDA approves patisiran to treat hereditary transthyretin amyloidosis. Nat. Rev. Neurol. 14 (10), 570. 10.1038/s41582-018-0065-0 30158559

[B146] Xiao L. Sakagami H. Miwa N. (2020). ACE2: The key molecule for understanding the pathophysiology of severe and critical conditions of COVID-19: Demon or angel? Viruses 12 (5), 491. 10.3390/v12050491 32354022 PMC7290508

[B147] Younis M. A. Tawfeek H. M. Abdellatif A. A. Abdel-Aleem J. A. Harashima H. (2022). Clinical translation of nanomedicines: Challenges, opportunities, and keys. Adv. Drug Deliv. Rev. 181, 114083. 10.1016/j.addr.2021.114083 34929251

[B148] Zhang G. Sun J. (2021). Lipid in chips: A brief review of liposomes formation by microfluidics. Int. J. Nanomedicine 16, 7391–7416. 10.2147/ijn.s331639 34764647 PMC8575451

[B149] Zhang X. Goel V. Robbie G. J. J. T. J. o. C. P. (2020). Pharmacokinetics of Patisiran, the first approved RNA interference therapy in patients with hereditary transthyretin‐mediated amyloidosis. J. Clin. Pharma. 60 (5), 573–585. 10.1002/jcph.1553 PMC7187331 31777097

[B150] Zhang Y. Almazi J. G. Ong H. X. Johansen M. D. Ledger S. Traini D. (2022). Nanoparticle delivery platforms for RNAi therapeutics targeting COVID-19 disease in the respiratory tract. Int. J. Mol. Sci. 23 (5), 2408. 10.3390/ijms23052408 35269550 PMC8909959

[B151] Zhang Y. Sun C. Wang C. Jankovic K. E. Dong Y. (2021). Lipids and lipid derivatives for RNA delivery. Chem. Rev. 121 (20), 12181–12277. 10.1021/acs.chemrev.1c00244 34279087 PMC10088400

[B152] Zhang Y. Xie F. Yin Y. Zhang Q. Jin H. Wu Y. (2021). Immunotherapy of tumor RNA-loaded lipid nanoparticles against hepatocellular carcinoma. Int. J. Nanomedicine 16, 1553–1564. 10.2147/ijn.s291421 33658783 PMC7920588

[B153] Zhang Z. Li J. Gu J. Amini R. Stacey H. D. Ang J. C. (2022). A universal DNA aptamer that recognizes spike proteins of diverse SARS‐CoV‐2 variants of concern. Chem. – A Eur. J. 28 (15), e202200524. 10.1002/chem.202200524 PMC9086990 35218097

[B154] Zhi D. Bai Y. Yang J. Cui S. Zhao Y. Chen H. (2018). A review on cationic lipids with different linkers for gene delivery. Adv. Colloid Interface Sci. 253, 117–140. 10.1016/j.cis.2017.12.006 29454463

[B155] Zhi D. Zhang S. Cui S. Zhao Y. Wang Y. Zhao D. (2013). The headgroup evolution of cationic lipids for gene delivery. Bioconjug Chem. 24 (4), 487–519. 10.1021/bc300381s 23461774

[B156] Zhi D. Zhang S. Wang B. Zhao Y. Yang B. Yu S. (2010). Transfection efficiency of cationic lipids with different hydrophobic domains in gene delivery. Bioconjug Chem. 21 (4), 563–577. 10.1021/bc900393r 20121120

[B157] Zhou J. Zhao Y. Simonenko V. Xu J. J. Liu K. Wang D. (2017). Simultaneous silencing of TGF-β1 and COX-2 reduces human skin hypertrophic scar through activation of fibroblast apoptosis. J. Oncotarget 8 (46), 80651–80665. 10.18632/oncotarget.20869 29113333 PMC5655228

